# Pancreatic ductal adenocarcinoma: integrating molecular insights for targeted interventions

**DOI:** 10.1038/s41392-026-02705-5

**Published:** 2026-05-05

**Authors:** Ganji Purnachandra Nagaraju, Haasita Nellipudi, Chaithanya Ganji, Vaishnavi Kuppala, Swapna Priya Ganji, Seema Kumari, Batoul Farran, Mundla Srilatha, Rachael Guenter, Bassel F. El-Rayes

**Affiliations:** 1 Division of Hematology and Oncology, Heersink School of Medicine, University of Alabama at Birmingham, Birmingham, AL USA; 2 Division of Medical Oncology, Moores Cancer Center, UC San Diego, La Jolla, CA USA; 3 School of Science, McMaster University, Hamilton, ON Canada; 4 Department of Biotechnology, Dr. B.R. Ambedkar University, Srikakulam, AP India; 5 Department of Hematology/Oncology, Henry Ford Health, Detroit, MI USA; 6 Department of Biotechnology, Sri Venkateswara University, Tirupati, Andhra Pradesh India; 7 Department of Surgery, Heersink School of Medicine, University of Alabama at Birmingham, Birmingham, AL USA

**Keywords:** Cancer, Oncology

## Abstract

Pancreatic ductal adenocarcinoma (PDAC) is one of the most lethal and aggressive tumor types, with a dismal 5-year survival rate of less than 15%. Despite major advances in understanding PDAC biology, therapeutic progress has been limited. Numerous preclinical studies have provided encouraging evidence that immune-based therapies may be effective. However, the clinical translation of immunotherapies for PDAC treatment has proven difficult with a lack of favorable tumor responses outside of a very select group of patients such as patients with MSI high tumors. Immune checkpoint inhibitors, as well as combination strategies with targeted radiotherapy or chemotherapy, have largely failed to demonstrate meaningful survival benefits for the majority of PDAC patients. Increasing evidence indicates that PDAC harbors a uniquely complex and multifaceted immunosuppressive microenvironment, which plays a central role in shielding malignant cells from effective antitumor immunity. Overcoming this barrier requires the development of rational and effective combination regimens that simultaneously target both the tumor and its surrounding immune microenvironment. Novel strategies, including the use of natural killer cell–based therapies, reprogramming of cancer-associated fibroblasts, and integration of predictive or prognostic biomarkers, hold promise for enhancing therapeutic efficacy. This review summarizes recent progress in PDAC immunotherapy, highlights key challenges, and discusses emerging approaches designed to improve patient outcomes.

## Introduction

Pancreatic ductal adenocarcinoma (PDAC) is an aggressive metastatic malignancy with a poor five-year survival rate (15%).^1^ Despite the recent advances in cancer treatment, PDAC remains notoriously resistant to most conventional therapies such as chemotherapy, highlighting the urgent need for novel approaches specifically tailored to this disease. While immunotherapy has achieved major successes in various cancers, immune checkpoint inhibitors (ICIs), alone or in combination with chemotherapy, have so far failed to improve outcomes in PDAC.^2–4^ This resistance to most forms of treatment stems from PDAC’s complex microenvironment (ME), characterized by a thick, dense stroma composed of diverse immune and stromal populations.^5^ Understanding the composition of the PDAC-ME and the complex interactions among its components is thus a prerequisite for overcoming its mechanisms of resistance and developing effective immune-based therapeutic solutions.

The immune populations present in PDAC-ME include effector T cells, which mediate anti-tumor immune responses, and regulatory T cells (Tregs), which exert immunosuppressive effects that inhibit anti-tumor immune responses.^6^ MDSCs (Myeloid derived suppressor cells) also promote the generation of an immunosuppressive ME that fosters tumor growth and drug resistance in PDAC.^7^ TAMs (Tumor associated macrophages), which can be polarized into the immunosuppressive M2 phenotype, further contribute to PDAC development and treatment refractoriness.^8^ Elucidating the exact roles of these populations and the pathways that govern their interactions in the PDAC-ME will thus inform the progress of novel therapeutic approaches capable of suppressing these mechanisms of resistance and enhancing therapeutic and survival outcomes in PDAC.

This review provides a detailed overview of the latest advances in immunotherapy in PDAC with focus on genetic and epigenetic regulations, targetable signal pathways and challenges in emerging biomarkers. The review examines the roles of the various populations within the PDAC-ME, including cancer associated fibroblasts (CAF), immune-promoting and suppressive cells. Additionally, the review summarizes ongoing clinical trials, thus shedding light on the latest advances and innovations in PDAC treatment.

## Overview of the PDAC-ME

The ME of PDAC is characterized by local inflammation, in which various immune cell subpopulations, including MDSCs, TAMs, and T cells, interact with CAFs and PDAC cells to promote inflammation and cancer progression. Stromal components, such as CAFs also modulate critical immune pathways that promote immune evasion.^9^ The stroma’s role in PDAC development is multifaceted and heterogeneous.^10^ In fact, the effects of different immune subsets are highly context-dependent, and influenced by variable factors such as disease stage (for example, precursor stage versus PDAC) or cell type.^11^ Hence, inhibiting particular stromal components at a given point during tumor progression could yield positive tumor control outcomes, but nonselective inhibition of several stromal elements may result in tumor progression. Furthermore, inhibiting a particular downstream pathway may promote tumor killing in a given cellular population but enhance tumor progression in another subset.^11^ Understanding the intricate interactions between different cell populations within the PDAC-ME could inform the advancement of more effective immunotherapy strategies.^4^ In the next section, we will describe various types of cancer-associated fibroblasts (CAFs) and immune cell populations in PDAC-ME, and their roles in PDAC immune suppression and evasion.

## PDAC-ME cells

### CAFs

**Fig. 1:**
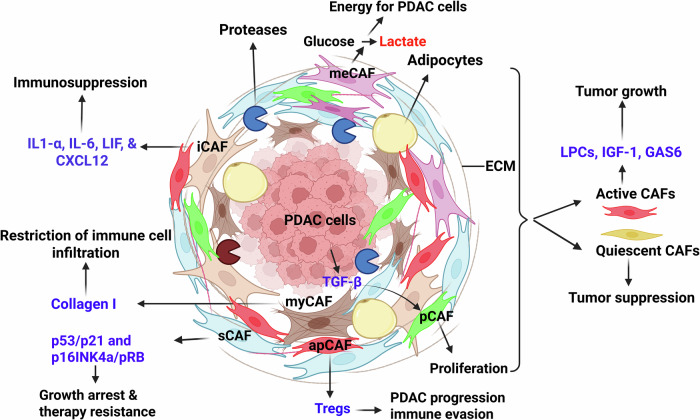
The microenvironment of PDAC. In PDAC extracellular matrix (ECM), quiescent CAFs (qCAFs), senescent CAFs (sCAFs), inflammatory CAFs (iCAFs), proliferative CAFs (pCAFs), metabolic CAFs (meCAFs), antigen-presenting (apCAFs), and myofibroblastic CAFs (myCAFs) are the seven main types of cancer-associated fibroblasts (CAFs). qCAFs secrete decorin (DCN), suppressing PDAC growth and metastasis. sCAFs are involved in PDAC growth inhibition and therapy resistance through overexpression of p53/p21 and p16INK4a/pRb. pCAFs contribute significantly to the fibrotic stroma in ME by TGF-β (secreted by PDAC cells). iCAFs promote immunosuppression by secreting chemokines and cytokines. meCAFs metabolize glucose to lactate, producing energy for PDAC growth. myCAFs produce collagen I, a significant part of the ECM, which limits the penetration of immune cells. apCAFs are involved in Tregs production, which is essential for immune evasion and PDAC progression. CAFs possess a dual role in both tumor-promoting and anti-tumor functions. They can stimulate PDAC growth by secreting insulin-like growth factor 1 (IGF-1), lysophosphatidylcholines (LPCs), and growth arrest-specific protein 6 (GAS6). Created in BioRender.com

PDAC is a complex disease involving various ME cell types, such as CAFs.^12^ CAFs can be derived from bone marrow mesenchymal stem cells or from activated pancreatic stellate cells (PSCs).^13^ CAFs play a diverse and vital role in the oncogenesis of PDAC, in tumor responsiveness to immune and conventional therapies, and in interactions with other key immune cell types. Distinct CAF subsets have been identified, each with unique characteristics and function (Fig. 1). The delineation and identification of different CAF subsets have been achieved using a variety of techniques. For instance, Öhlund et al.^14^ applied spatial and functional tests to differentiate inflammatory CAFs (iCAFs) and myofibroblastic CAFs (myCAFs) based on their proximity to tumor cells and cytokine production, whereas Elyada et al.^15^ identified antigen-presenting CAFs (apCAFs) via single cell sequencing (scRNA-seq) and MHC class II expression. Senescent CAFs (sCAFs) play complex roles in PDAC growth, immune responses, and therapy resistance. Within the broader CAF landscape, myCAFs primarily remodel the extracellular matrix (ECM), whereas iCAFs promote an immunosuppressive milieu. In contrast, quiescent CAFs (qCAFs) do not express or secrete inflammatory factors, cytokines, or growth factors that contribute to PDAC progression. Metabolic CAFs (meCAFs) support tumor growth and survival by shifting their metabolic profiles, and antigen-presenting CAFs (apCAFs) can present antigens to immune cells, shaping PDAC–immune interactions. Finally, proliferative CAFs (pCAFs) display enhanced proliferation, driven by signaling pathways activated by PDAC-ME signals. These CAF subsets can be defined by distinct gene marker expression profiles (Table 1). Moreover, different CAF subsets can interact with other major immune cell types, including MDSCs and TAMs. It is important to note that Carpenter et al.^16^ recently highlighted that CAF subset designations, while important, are frequently context-dependent and can vary with the analytical platform and ME signals. The overall CAF–MDSC–TAM axis plays an important role in shaping the immunosuppressive microenvironment in PDAC. This axis features bidirectional relationships between the immune cell types to amplify immunosuppressive feedback loops. In the context of PDAC, CAFs regulate tumor progression by remodeling the ECM, creating immunosuppressive niches, modulating metabolism, presenting antigens (Ags), influencing resistance to therapy, and coordinating crosstalk with immune cells such as MDSCs and TAMs within the ME.^17^ Understanding these different CAF subsets and their interactions with other immune cell types is important for developing targeted therapeutic strategies for PDAC.

**Table 1:** Biomarkers for identification of cancer associated fibroblasts

qCAFs (PSCs)	sCAFs	iCAFs	myCAFs	meCAFs	pCAFs	apCAFs
α-SMA↑	lL- 1α↑	lL-6↑	Vimentin↑	Bnip3↑	Stmn1↑	Cd74↑
FAP↓	lL- 1β↑	lL-8↑	FAP↑	Slc2a1↑	Top2A↑	Slpi↑
Dcn↑	lL-6↑	Cxcl12↑	PDPN↑	Hilpda↑	Nusap1↑	Saa3↑
	lL-8↑	α-SMA↓	α-SMA l	Vegfa↑	Cenpf↑	H2-ab1↑
	TGF- β↑	FAP l		Ndrg1↑		

↑indicate high;↓ indicate low; l indicate average

#### Proliferative CAFs (pCAFs)

pCAFs represent a distinct subgroup within the ME, particularly notable in PDAC.^18^ This CAFs subset has a higher proliferative capacity than normal fibroblasts, contributing significantly to the fibrotic stroma of PDAC.^18^ PDAC cells and ME components produce cytokines and growth factors like TGF-β (transforming growth factor-beta), PDGF (platelet-derived growth factor), and FGF (fibroblast growth factors) that induce paracrine proliferative effects.^19^ pCAFs then respond to these signals by upregulating various signaling pathways, including TGF-β/SMAD, PI3K/Akt, MAPK (ERK1/2, JNK, p38), and STAT3, which collectively promote a pro-tumorigenic microenvironment.^19,20^ TGF-β is one of the most widely implicated drivers of CAF activation and expansion in PDAC (Fig. 1), with elevated levels reflecting its increased production and release by both tumor and stromal cells.^19^ TGF-β promotes CAF activation through canonical SMAD-dependent signaling, as well as non-canonical pathways, collectively supporting myofibroblastic features, matrix remodeling, and pro-tumor stromal functions.^21^ The activation of CAFs through the interaction of TGF-β and SMAD signaling induces the phosphorylation of SMAD2/3, which forms a complex with SMAD4 that then translocates to the cell nucleus to regulate transcription of fibroblast activation genes such as α-SMA, collagen, and fibronectin.^22^ TGF-β/SMAD CAF activation consequently promotes a myofibroblastic CAF phenotype. Examples of non-canonical signaling cascades activated by TGF-β include MAPK (ERK, JNK, p38) and PI3K/AKT/mTOR pathways, which can both enhance fibroblast proliferation and survival while stimulating the secretion of pro-tumorigenic cytokines.^23–25^ TGF-β also intersects with other signaling networks, including Wnt/β-catenin, Notch, and Hedgehog, to further amplify fibroblast activation and matrix deposition.^26^ Overall, TGF-β plays a key role in CAF biology by regulating ECM remodeling, tumor-supportive cytokine secretion, and immune evasion within the PDAC microenvironment through its interaction with critical cell signaling pathways.

Another key growth factor that promotes CAF proliferation is PDGF. PDGF is produced by tumor cells and other stromal cells and signals through PDGF receptors on CAF surfaces.^27^ Upon receptor binding, PDGF activates downstream intracellular pathways that support CAF growth and survival. Beyond expanding CAF numbers, PDGF signaling also helps maintain CAF activation and reinforces their pro-tumorigenic functions within the tumor microenvironment. The characterization of CAFs in PDAC reveals that CAF biomarkers (e.g., α-SMA, FSP1, FAP, PDGFR-α/β) lack cell-type specificity and exhibit dynamic, context-dependent expression.^28^ This is partly driven by sustained PDGF signaling that maintains CAF activation and pro-tumorigenic functions within the TME, resulting in pronounced CAF heterogeneity and plasticity.

FGF is another critical CAF-interacting growth factor. FGF can induce cell proliferation and is dysregulated in various cancers, including PDAC.^29^ FGF signaling induces the synthesis of growth factors and cytokines to promote CAF proliferation, either directly or indirectly.^30^ In addition, FGF can affect the ME by inducing angiogenesis, thereby providing indirect support for CAF proliferation and survival.^31^

pCAFs secrete a broad range of chemokines, growth factors, and cytokines that reinforce their own proliferation and survival while enhancing PDAC cell invasion, factors that collectively accelerate tumor progression.^32^ pCAFs play a key role in the pathogenesis of PDAC and may constitute new targets for therapy. Strategies targeting pCAFs or disrupting the pathways they activate could reduce the fibrotic stroma to improve therapeutic efficiency in PDAC. Further investigation of the reciprocal crosstalk between pCAFs and PDAC cells is needed to clarify how it shapes tumor heterogeneity and contributes to therapeutic resistance in PDAC.

#### Myofibroblastic CAFs (myCAFs)

myCAFs represent an integral part of the PDAC-ME and play several roles during tumor progression and metastasis.^33^ myCAFs are characterized by elevated expression of alpha smooth muscle actin (α-SMA). Similar to pCAFs, myCAFs can also undergo activation via TGF-β dependent canonical (SMAD2/3) and non-canonical signal pathways.^33^ Following activation by signals from tumor or stromal cells, myCAFs significantly contribute to remodeling of the ECM within the PDAC stroma.^33^ They produce different types of ECM components, including collagen, fibronectin, and proteoglycans, which help form the heavy, fibrotic stroma pathognomonic desmoplastic feature of PDAC (Fig. 1). Alterations in the tumor ECM composition confer structural integrity for the tumors and generates physical and biochemical barriers that impede drug delivery and immune cell invasion and activation into the PDAC parenchyma.^34^ Through this ECM alteration, myCAFs can promote resistance to therapies against PDAC. In addition to their roles in ECM remodeling, myCAFs can also promote cancer cell invasion, migration, and metastasis.^33^ They secrete various growth factors, cytokines, and proteases that can facilitate the degradation of the basement membrane and ECM, thus enhancing the motility and invasion of tumor cells into adjacent tissues.^35^ Additionally, myCAFs can directly interact with cancer cells to induce epithelial to mesenchymal transition (EMT) and metastasis in PDAC.^36^ Through paracrine and juxtacrine signaling, myCAFs directly interact with PDAC cells to promote the EMT.^37^ Adhesion-associated signaling, such as integrins, cadherins, and mechanotransduction signalings including FAK/Src, and YAP/TAZ, are triggered by physical contact between CAFs and PDAC cells, and EMT-related transcriptional programs are reinforced by TGF-β, IL-6, and CXCL12, which are secreted from CAFs.^38,39^ These convergent molecules induce tumor cell plasticity, invasion, and resistance to treatment by reducing E-cadherin (an epithelial marker) and upregulating SNAIL, SLUG, and ZEB1 (mesenchymal regulators).^14,40^ The function of myCAFs in tumors must be carefully considered in a context-dependent fashion. It has been reported that myCAFs can act as tumor-restraining barriers in early disease phases, while promoting tumor progression, immune exclusion, and therapy resistance at advanced stages in PDAC.^41^

#### Inflammatory CAFs (iCAFs)

iCAFs are another subgroup of CAFs that play an essential part in the PDAC-ME.^42^ The secretion of various inflammatory mediators distinguishes the association of CAFs with inflammation. iCAFs can express various cytokines, including interleukins and TNF-α, as well as chemokines and prostaglandins, in response to signals from tumor cells and the surrounding ME.^43^ Secretion of these factors can result in a chronic inflammatory state, contributing to PDAC growth and progression.^44^ Furthermore, the secretion of chemokines, such as CXCL12, CCL8, and CXCL2 from iCAFs can act as chemoattractants for TAMs and neutrophils (Fig. 1). The influx of these immune cells exacerbates inflammation within the ME and further promotes PDAC progression.^45^ Immune cells recruited to the PDAC ME can produce factors such as IDO, PGE_2_, and TGF-β, that suppress the activity of T and NK cells while upregulating immunosuppressive cell populations,^9^ ultimately leading to immune escape. The inflammatory ME, supported by iCAFs, contributes to tumor proliferation, angiogenesis, and EMT, to enhance PDAC aggressiveness and metastatic potential.^9^ In summary, iCAFs shape the inflammatory PDAC-ME and drive tumor growth by interacting with both PDAC and immune cells.

#### Senescent CAFs (sCAFs)

sCAFs represent a subset of CAFs that influence PDAC growth and resistance to therapy.^46^ These cells are characterized by a state of permanent growth arrest mediated via pathways such as p53/p21 and p16INK4a/pRB. It is well established that the tumor suppressor protein p53 regulates cellular responses to stress and oncogene activation.^47^ DNA damage response leads to initiation of ATM/ATR kinases, which phosphorylate and stabilize p53. P53 induces p21 and senescence-associated proteins.^48^ Similarly, the p16INK4a/pRB pathway is another important regulator of cellular senescence.^49^ The protein p16INK4a inhibits the action of cyclin dependent kinases (CDK)-4 and −6 to block the phosphorylation of the pRB.^50^ Hypophosphorylated pRB then inactivates E2F, a factor essential for cell cycle succession.^51^ In summary, cell cycle arrest and senescence induction of sCAFs can be activated through p53/p21 or p16INK4a/pRB pathways to cause these cells to lose proliferative capacity within the tumor stroma (Fig. 1).^51^

Although senescent, sCAFs can secrete various factors known as Senescence-Associated Secretory Phenotype (SASP), comprising growth factors, chemokines, pro-inflammatory cytokines, ECM remodeling enzymes, and proteases.^52^ SASP influences neighboring cancer cells and stromal components, thereby modulating tumor growth, invasion, and therapy response. Specifically, interleukin (IL)-6 and −8 promote PDAC cell proliferation and angiogenesis, while proteases facilitate tumor cell invasion via ECM degradation.^53^ Furthermore, SASP creates a pro-survival ME that fosters therapy resistance by enhancing cancer cell survival post-treatment and impeding drug penetration into the tumor. In conclusion, sCAFs have emerged as crucial contributors to PDAC pathogenesis and pose challenges for therapeutic intervention.^46^ Understanding their role in tumor-stroma interactions is essential for devising effective strategies to combat this aggressive malignancy.

#### Metabolic CAFs (meCAFs)

meCAFs exhibit distinct metabolic profiles compared to normal fibroblasts, characterized by increased aerobic glycolysis (the Warburg effect) and altered nutrient uptake and utilization.^36^ Metabolic rewiring enables CAFs to support tumor growth and survival by providing energy and essential building blocks for cancer cells. One of the most prominent features of meCAFs is their reliance on aerobic glycolysis, wherein glucose is preferentially metabolized to lactate (Fig. 1).^54^ This metabolic shift generates energy for meCAFs and produces lactate, which can be used by adjacent PDAC cells as a fuel source. Moreover, metabolic CAFs exhibit heightened glycolytic flux, increasing glucose uptake and lactate production, further contributing to the nutrient-rich ME.^54^

meCAFs also undergo alterations in nutrient consumption and utilization, including differential uptake of lipids and amino acids, which support pancreatic tumor growth and development.^54^ In PDAC, meCAFs have been shown to drive tumor progression through regulation of interconnected signaling networks such as TGF-β, Hedgehog, Notch, Wnt (β catenin), Hippo, NF-κB, JAK/STAT, MAPK, and PI3K/AKT within the PDAC-ME.^55^ Transcription factors (TFs) with oncogenic roles, such as HIF, HSF1, p53, Snail, and Twist, have been shown to regulate meCAF phenotypic plasticity.^56^ Altogether, these metabolic changes and the interaction of meCAFs with other signaling pathways support a tumor-promoting role of meCAFs in PDAC, creating a collaborative environment that enables PDAC cells to grow, survive, and invade through metabolic symbiosis. Further elucidation of the metabolic interrelationship between PDAC cells and meCAFs could provide the conceptual framework for targeting therapeutic interventions that disrupt such metabolic dependencies to arrest tumor growth in PDAC.

#### Antigen presenting CAFs (apCAFs)

apCAFs are a functionally distinct immunoregulatory stromal population in PDAC that shapes cell-mediated immune responses within the TME.^57^ These cells have the distinctive capacity to acquire, process, and present antigens (Ags) to immune cells, especially T cells.^58^ Such Ag presentation occurs through antigenic peptides on MHC molecules, including MHC class I and II, expressed on the membrane of CAFs.^58^ apCAFs are critical for initiating and regulating immune responses against tumors through their interactions with T cells, thereby affecting tumor development. By modulating variation, function, and activity, CAFs may orchestrate proinflammatory responses due to cytotoxic T cell activation targeting PDAC cells or immunosuppressive responses (Fig. 1).^59^ Further, apCAFs can engage in cross-presentation by internalizing Ags derived initially from other cancer cells and stromal cells, thus further enhancing immune responses toward tumor Ags. apCAFs can also interact with tumor-infiltrating CD4⁺ T cells and are enriched in immune checkpoint blockade (ICB) sensitive cases of PDAC.^57^ apCAFs activate effector CD4⁺ T cells while simultaneously driving regulatory T-cell (Treg) differentiation, suggesting a dual and context-dependent role.^60^ Moreover, apCAF depletion in immunosensitive tumors diminishes responsiveness to ICB, proving apCAFs as essential contributors to effective antitumor immunity.^61^ Transcriptomic profiling further reveals that apCAF-induced Tregs in ICB-resistant tumors exhibit enhanced expression of immunosuppressive gene programs, driven by chemokine signaling from apCAFs.^62^ Given their crucial role in tumor immunity, immunotherapeutic approaches focusing on apCAFs have received considerable attention.^59^ Several approaches, such as delivering immunomodulatory agents to CAF-specific receptors or engineering CAFs to express co-stimulatory molecules, have been developed to harness their antigen-presenting function, thereby improving treatment outcomes in PDAC.

### MDSCs

MDSCs represent a heterogeneous group of myeloid cells capable of suppressing innate and adaptive immunity via different mechanisms. These pathways, including the production of NO, ARG1, and ROS, deplete the essential amino acids (cysteine and arginine) vital for T-cell activation, proliferation, and function.^63^ MDSCs also contribute to the induction of T-regs, TAMs (tumor-associated macrophages), and B-regs, thereby increasing immune suppression within the PDAC-ME.^64^ Two subtypes of MDSCs have been defined: polymorphonuclear MDSCs (PMN-MDSCs) and monocytic MDSCs (M-MDSCs), both of which contribute to immune-evasion in PDAC.^65^ The activation of M-MDSCs triggers VEGF (vascular endothelial growth factor) production,^66^ which enhances angiogenesis and further perpetuates an immunosuppressive ME. High VEGF levels contribute to the depletion of essential amino acids required for the immune cells proliferation, thus promoting suppression of anti-tumor immune responses.^67^ PMN-MDSCs represent a unique cellular subset within the highly complex PDAC-ME immune landscape.^68^ Interference with T cell functions is one of the mechanisms by which PMN-MDSCs establish an immune-suppressive environment, which in turn favors tumor progression. Both subtypes of MDSCs respond to signals derived from the PDAC-ME, including factors such as VEGF.^69^ In summary, MDSCs contribute to the immunosuppressive environment in PDAC by influencing mechanisms of tumor progression and limiting therapeutic efficiency.^64^

MDSCs also impact PDAC progression through their interaction with CAFs within the PDAC ME. The CAF-MDSC axis enables PDAC to build an immunosuppressive niche. CAFs can promote MDSCs through multiple mechanisms, including recruitment via chemokines and differentiation and expansion by cytokines and growth factors. CAFs, predominantly iCAFs, secrete chemokines such as CCL2, CXCL5, and CXCL12, which in turn recruit MDSCs into the tumor or trigger monocytes to differentiate into monocytic MDSCs.^44^ MDSCs can also be recruited into the tumor, acquiring an immunosuppressive phenotype through cytokines and growth factors secreted by CAFs. IL-6 and TGF-β1 have each been shown to promote the conversion of myeloid cells into immunosuppressive MDSCs.^70,71^ In PDAC, the CAF-MDSC axis supports immunosuppression through STAT3 activation in MSDCs due to IL-6 derived from CAFs.^72^ It is important to note that the CAF-MDSC axis can operate as a feedback loop. Not only can CAFs have activity that recruits and differentiates MDSCs, but MDSCs can also produce factors that maintain or reprogram fibroblasts into activated CAF states, such as iCAFs. targeting the interactions between MDSCs, iCAF, and other components of the PDAC-ME could be for a rational approach for developing novel effective therapeutic strategies that overcome immune resistance.

One specific mechanism by which MDSCs foster an immunosuppressive ME within a tumor is the upregulation of TMBIM1 (transmembrane BAX inhibitor motif-containing 1) in PDAC cells, which, in turn, enhances MDSC recruitment and infiltration.^73^ Targeting TMBIM1 may be a therapeutic approach for alleviating MDSC-mediated immune suppression in PDAC. Liu et al.^74^ identified another novel pathway by which tumor cells can potentiate MDSCs. They revealed that PDAC cells upregulate Dickkopf-1 (DKK1) expression in nerves, promoting MDSCs infiltration. Conversely, DKK1 inhibition reduced MDSC levels, impairing MDSC-mediated immune suppression. These findings widen the horizons of tumor-ME research by demonstrating that a nerve-released protein can modulate the PDAC-ME, thus establishing a nerve-PDAC axis and uncovering new layers of complexity in tumorigenesis. Despite these major strides in our understanding of PDAC, the pathways mediating the differentiation of neutrophils into polymorphonuclear-MDSCs (PMN-MDSCs), which drive cancer aggressiveness, remain unknown. Elucidating these pathways is thus key to uncovering new therapeutic targets and improving immunotherapy responses in PDAC patients.^75^ A novel study showed that cancer-derived lipid mediators, located close to the tumor niche, can promote the differentiation of neutrophils into MDSCs, thus contributing to immunosuppression in PDAC.^76^ The study also revealed that the inflammatory molecule PAF (platelet activation factor) can polarize neutrophils towards immunosuppressive phenotypes. This novel mechanism of MDSC differentiation may represent a potential immunotherapeutic strategy for PDAC and other solid tumors. Another study investigated the role of CRIP1, cysteine-rich intestinal protein 1, in PDAC progression and ME re-programming.^77^ Elevated CRIP1 levels enhanced MDSCs infiltration, resulting in an immunosuppressive ME. Mechanistic investigation showed that CRIP1 binds to NFκB, leading to its nuclear translocation and subsequent CXCL1/5 activation, which can promote MDSC migration. Inhibition of this pathway suppressed MDSC recruitment and trafficking and enhanced immunotherapy, highlighting the therapeutic potential of this pathway for PDAC treatment.

Sun et al.^78^ showed that CXCR4 CAR-T cells exhibit enhanced anti-tumor efficacy in vivo. These CAR-T cells exerted their effects by impairing STAT3 signaling, thus reducing the release of inflammatory cytokines such as IL-6 and TNFα. This, in turn, suppressed the release of SDF-1α by CAFs through NFκB signaling, thus hampering the migration of MDSCs to tumor sites. These findings suggest that CXCR4 + CAR-T cells may be a promising therapeutic strategy for PDAC, although further studies are required to corroborate their efficacy. Kajiwara et al. evaluated the effectiveness of a telomerase-specific oncolytic adenovirus armed with p53 gene (OBP-702), in gemcitabine (Gem)-resistant PDAC cells.^79^ The observation that in the ME of Gem-refractory PDAC increased levels of PDL-1 produced increased levels of GM-CSF, resulting in MDSC accumulation and immunosuppression. OBP-702 inhibited GM-CSF-associated MDSC accumulation and enhanced the anti-tumor effects of PDL-1 treatment in these cells. Thus, combining OBP-702 and anti-PDL-1 therapy may be promising for treating Gem-resistant PDAC tumors. Future research will assess the feasibility and efficacy of MDSC-based immunotherapies for the treatment of PDAC.

### Tumor associated macrophages (TAMs)

TAMs are abundant immune cell populations within the PDAC-ME.^80^ They display anti-inflammatory and pro-inflammatory properties and can further be polarized into M1 and M2 macrophages. In PDAC, the M2 subtype is predominant and exerts pro-tumoral effects. TAMs express ligands and receptors implicated in regulating immune responses in the PDAC-ME, and mediate their immunosuppressive functions through a multitude of mechanisms.^81^ For instance, TAMs express arginase-1 and IDO, which deplete essential amino acids needed by proliferating T cells,^82,83^ thus impairing cytotoxic T-cell function and generating an immunosuppressive tumor microenvironment. TAMs overexpress PD-L1, PD-1, and Human Leukocyte Antigen (HLA).^84^ These ligands act as inhibitory receptors for CD94, IL-2, and IL-4, facilitating immune escape and exhaustion of T-cells, and further weakening the anti-PDAC immune response.^85,86^ In a recent study published in Nature, Caronni et al.^87^ employed spatial genomics and single-cell analysis in combination with functional studies to investigate the role of macrophages in PDAC. The group found that a subset of macrophages termed IL-1β + TAMs, characterized by inflammatory programs, promotes PDAC progression. Tumor-infiltrating monocytes differentiate to IL-1β^+^ TAMs following exposure to TNF and PGE2, leading to inflammatory reprogramming of adjacent PDAC cells. This, in turn, promotes the production of TNF, PGE2, and other factors, creating a positive feedback loop that reinforces IL-1β phenotypes. IL-1β TAMs interact with PDAC cells expressing an IL1β specific response, T1RS, linked to poor patient outcomes. Additionally, IL-1β TAMs are enriched in hypoxic tumor niches, where tissue repair, immune suppression, and inflammation co-exist. This study suggests that inflammatory reprogramming may occur early during PDAC tumorigenesis, resulting in transcriptional modifications associated with cancer progression and poor outcomes. IL-1β or PGE2 inhibition suppressed inflammation in the PDAC milieu and hampered PDAC progression,^87^ indicating that IL-1β^+^ TAMs may be a potential therapeutic target in PDAC. To elucidate the role of TAMs in PDAC progression, Tabe et al.^88^ developed a PDAC organoid incorporating TAMs. The model successfully mimicked the heterogeneity of TAM and CAF populations in PDAC, recapitulating the complex PDAC ME. The diverse TAM subtypes enhanced angiogenesis and tumor survival, suggesting that TAMs may promote vascular structure expansion during PDAC growth. Another study found that TAMs could promote PDAC tumorigenesis by boosting glycolysis via the IL-8/STAT3/GLUT3 signaling.^89^ An innovative study by Xu et al.^90^ uncovered the role of a new UPR sensor, termed CREB3L1, in PDAC progression. CREB3L1 is an ER stress transducer that localizes to the ER in its inactive state. CREB3L1 activation leads to its nuclear translocation, initiating the transcription of target genes such as Xbp1 or Col1a1. Xu et al.^90^ found that CREB3L1 is upregulated in PDAC and can remodel the TME by polarizing macrophages towards immunosuppressive M2 phenotypes. This in turn impairs the antitumor activity of cytotoxic TILs, leading to immunotherapy resistance. Targeting the pathways through which TAMs elicit drug resistance will thus help improve treatment outcomes in PDAC patients.

Another mechanism through which TAMs may promote PDAC aggressiveness is through exosome transfer. A recent study found that PDAC patient TAMs enhanced tumor migration and invasion through exosomes.^91^ This effect was specifically mediated by miR-142-5p and miR-202-5p, which were transferred to PDAC cells through exosomes released by TAMs, leading to PTEN suppression and enhanced invasion. Hence, targeting exosomes may be a potential strategy for alleviating metastasis and immune evasion in PDAC.

TAMs orchestrate a multilayered immunosuppressive program in the PDAC-ME, involving metabolic depletion of essential nutrients, expression of ICMs (immune checkpoint molecules), angiogenesis, crosstalk with the stroma, exosome transfer, and bidirectional interactions with MDSCs and CAFs.^92^ TAMs interact with both MDSCs and CAFs to promote an immunosuppressive, pro-tumorigenic ME.^92^ The TAM–MDSC part of the axis contributes to reinforcing myeloid immunosuppression. This happens through shared lineages and differentiation pathways, bidirectional activation triggers, and cytokine feedback loops. Monocytic MDSCs can serve as precursors of TAMs.^93,94^ In the ME, M-MDSCs can be exposed to various growth factors and metabolites that ultimately drive their differentiation into immunosuppressive M2-like TAMs.^7,95,96^ On the other hand, TAMs can secrete chemokines, cytokines, and growth factors that promote the expansion, polarization, and survival of MDSCs. Examples of MDSC-supporting factors secreted from TAMs are IL-10, TGF-β, and VEGF.^97^ TAM–CAF crosstalk contributes to stromal remodeling and immune evasion via CAF-induced macrophage recruitment and polarization, as well as TAM-mediated CAF activation and maintenance. CAFs can secrete chemokines and cytokines, which can drive circulating monocytes toward an M2-like TAM phenotype.^95,98^ TAMs can reciprocally support CAFs through the production of factors such as TGF-β, PDGF, and IL-1β that activate CAFs.^44^ Overall, the complexity of the CAF-TAM–MDSC axis maintains the immunosuppressive ME of PDAC and may also explain therapy resistance. Targeting these specific interactions could inform novel therapeutic avenues aimed at restoring and enhancing anti-tumor immunity in PDAC.

### Dendritic cells (DCs)

**Fig. 2:**
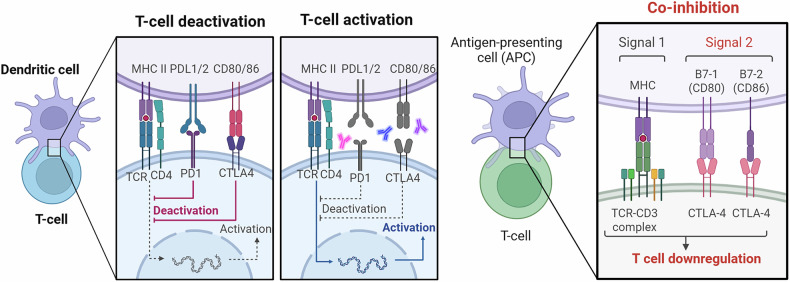
T-cell activation, deactivation, and co-inhibition are regulated by immune checkpoints. The balance between activating and inhibitory signals controlling T-cell responses during antigen presentation is depicted in this schematic. Dendritic cells and other antigen-presenting cells (APCs) provide Signal 2 through co-stimulatory interactions between CD28 on T cells and B7 family ligands (CD80/CD86) on APCs, which promote T-cell activation and transcriptional programs linked to effector function, and Signal 1 through engagement of the T-cell receptor (TCR)–CD3 complex with peptide–MHC complexes. On the other hand, immune checkpoint receptors, such as PD-1 and CTLA-4, bind PD-L1/PD-L2 and CD80/CD86, respectively, thereby either competing with or overriding co-stimulatory pathways. T-cell deactivation or functional exhaustion decreased cytokine production, and attenuation of TCR signaling is the outcome of dominance of these inhibitory interactions. The right panel illustrates CTLA-4-mediated co-inhibition, in which T-cell downregulation results from CTLA-4’s interaction with B7 ligands, limiting co-stimulatory signaling downstream of the TCR–CD3 complex. The figure, as a whole, emphasizes that T-cell fate and immune responsiveness are determined by the dynamic interplay between activating and inhibitory checkpoints, a fundamental concept in immune homeostasis and cancer immunotherapy. Created in BioRender.com

The dense stroma of PDAC can obstruct the infiltration of DCs and other immune cells into the tumor parenchyma, thus compromising DC function.^99^ The stromal barrier, which consists of fibroblasts, ECM proteins, and immune cells, limits the access of DCs to tumor Ags, preventing the start of an effective antitumor immune response.^100^ Additionally, the immunosuppressive PDAC milieu releases TGF-β and VEGF, which reduces DC function.^101^ For instance, TGF-β inhibits DC maturation, reducing the capacity of DCs to present Ags to T cells and initiate immune responses (Fig. 2). TGF-β also blocks the co-stimulatory expression molecules required to activate T cells on DCs.^102^ Conversely, VEGF promotes angiogenesis, supports tumor growth, and inhibits DC function by regulating DC differentiation and maturation, reducing their capacity to present Ags and trigger T cells.

Despite these challenges, the promising potential of DCs as immunotherapy targets in PDAC has led to the development of DC-based vaccines. This approach consists of pre-loading DCs with tumor Ags to stimulate anti-tumor immune responses in patients. Vaccines can overcome the immunosuppressive barriers posed PDAC by delivering tumor antigens directly to DCs and bypassing Ag processing within the ME.^103^ Various combination strategies, such as combined use of vaccines and ICIs or adjuvants that promote DC maturation, have been developed so far and are delineated in Fig. 2.

In addition, there is growing evidence that interactions between DCs and other immune cells within the ME influence not only PDAC progression but also its response to therapy. Therefore, there is a growing need to gain further insights into the sophisticated immune responses mediated by DCs and into the cross-talk among DCs, T cells, and other immune cells to design effective immunotherapeutic strategies tailored to PDAC (Fig. 1).^103^ Within the TME, the DCs can undergo profound lipid metabolic reprogramming characterized by enhanced fatty acid uptake, intracellular lipid accumulation, and increased oxidation of fatty acids.^104^ This metabolic shift impairs DC Ag processing and presentation capacity through mechanisms involving CD36-mediated lipid uptake, defective autophagy (Atg5 loss), and XBP1-driven ER stress, ultimately reducing the effectiveness of CD8⁺ T-cell priming (Fig. 2). Elevated fatty acid oxidation mediated by CPT1A promotes the secretion of immunosuppressive cytokines and facilitates regulatory T-cell recruitment through the Wnt5a–β–catenin–PPARγ signal pathway.^105^ Together, these alterations render DCs functionally tolerogenic and support tumor immune evasion.^106^ PDAC has shown impaired antitumor immunity due to deficient infiltration and function of conventional DC driven by low intra-tumoral Flt3L expression.^107^ Combined Flt3L and CD40 agonist therapy restores cDC abundance and activation, inducing a cDC1-dependent type I immune activation featured by increased CD8⁺ T-cell priming, IL-12 production, and reciprocal IFN-γ signal pathway.^107^ While this strategy improves T-cell infiltration and antitumor immunity in preclinical PDAC models, concurrent activation of cDC2s increases regulatory T-cell accumulation, signifying the therapeutic potential and the need for further refinement of DC-targeted immunotherapies.^107^ In summary, targeting DC-mediated defense responses and overcoming immunosuppressive barriers in the ME are promising strategies to improve the antitumor efficacy of immunotherapy and to advance outcomes in patients with PDAC.

## Lymphocytes and n atural killer cells

### T cells and their function in PDAC

The PDAC-ME presents a formidable barrier to effective antitumor immune responses, characterized by the secretion of several inhibitory factors that suppress T cell functions. As previously mentioned, PDAC is characterized by a dense desmoplastic stroma that forms physical barriers to T cell access within the tumor parenchyma (Fig. 2). The desmoplastic stroma is mainly composed of fibrotic tissue with plenty of ECM elements, including collagen and hyaluronic acid, CAFs, and immune cells.^108^ This compact stromal structure restricts the movement of active immune cells within the ME, such as T cells, thereby reducing contact between those active immune cell types and PDAC cells deeply embedded in the tumor mass.

Despite these challenges, a subset of T cells, in particular CD8^+^ T cells, can still infiltrate PDAC and exert antitumor effects.^109^ These T (CD8^+^) cells recognize tumor-specific Ags, which are exhibited by MHC signals on the membrane of PDAC cells. Upon identifying these Ags, CD8^+^ T cells become activated and exert cytotoxic activity to eliminate PDAC cells. However, the suppressive ME, characterized by factors like inhibitory cytokines, ICMs, and metabolic alterations, often undermines the role of these effector T cells. This immune inhibition causes T cell dysfunction and exhaustion, allowing PDAC immune avoidance and progression despite the infiltration of cytotoxic T cells (Fig. 2).^110^ Thus, overcoming the immune inhibition barriers within the PDAC environment is important for improving T cell centered immunotherapies and patient outcomes in PDAC treatment.

### T-cell activation, deactivation, and co-inhibition

Ag-presenting cells (APCs), such as DCs, integrate activating and inhibitory signals during antigen (Ag) presentation to tightly regulate T-cell activation (Fig. 2). T-cell receptor (TCR)–CD3 complex binding with peptide–MHC complexes (Signal 1) and co-stimulatory signaling, which is mainly mediated by CD28 binding to the B7 family ligands CD80 and CD86 on APCs (Signal 2), are necessary for productive T-cell activation.^111^ T-cell proliferation, cytokine production, and effector differentiation are all fueled by downstream transcriptional programs initiated by this coordinated signaling.^112^ To preserve immunological homeostasis and avoid overactivation, immune checkpoint receptors also play a crucial role as negative regulators of T-cell responses.^113^ Through distinct yet complementary mechanisms, cytotoxic T-lymphocyte–associated protein 4 (CTLA-4) and programmed cell death protein 1 (PD-1) attenuate T-cell signaling.^114^ PD-1 engagement by its ligands, while CTLA-4 limits co-stimulatory input by competing with CD28 for binding to CD80/CD86 with higher affinity, PD-L1 or PD-L2 suppresses proximal TCR signaling and metabolic activity (Fig. 2).^115^ When these inhibitory pathways predominate, the balance shifts toward T-cell deactivation, characterized by decreased transcriptional activity and compromised effector function. Targeting the PD-1/PD-L1 and CTLA-4 pathways in cancer immunotherapy is therapeutically justified because dysregulation of this checkpoint balance, especially in the tumor microenvironment, encourages immune evasion and T-cell dysfunction.

### T (CD4^+^) cells

Within the context of PDAC, T (CD4^+^) cells can function both in favor of and against the tumor, depending on multiple factors in the ME.^116^ One role of T (CD4^+^) cells in PDAC is to regulate immune responses by supporting T (CD8^+^) cells, B cells, and macrophages.^117^ Helper T cells are activated upon recognizing tumor Ag’s presented by APCs in the draining lymph nodes. Upon activation, CD4^+^ T cells differentiate into distinct subgroups, including T helper cells (Th1, Th2, and Th7) and Tregs, each with unique functions and cytokine profiles.^118^

Th1 cells produce TNF-α and IFN-γ, promoting the activation of CD8^+^ T cells and macrophages, which can directly kill PDAC cells. Th2 cells produce IL-4 and IL-13, inducing B cells to secrete antibodies and promote tumor-associated fibrosis.^118^ Th17 cells produce IL-17 and IL-22 and promote inflammatory responses and tissue injury in the ME. In contrast, Tregs suppress the immune response and promote immune tolerance, thereby weakening anti-PDAC immunity and promoting tumor immune escape.

This balance among these subsets of T (CD4^+^) cells is essential for determining the overall effect of the immune reaction against PDAC.^119^ Imbalance, which favors pro-tumorigenic subsets, including Th2 cells and Tregs, will promote PDAC through the inhibition of anti-PDAC immune reactions and the acceleration of immune evasion.^119^ In contrast, when anti-tumorigenic subsets, including Th1 cells, are abundant, there will be an increase in immunity and a reduction in PDAC growth. In PDAC, impaired functionality of DCs and limited Ag presentation lowers the CD4⁺ T-cell responses toward tolerogenic and immunosuppressive states, thus preventing effective antitumor response. In PDAC, increased DC infiltration inconsistently promotes tumor progression by specifically recruiting regulatory CD4⁺ T cells that disrupt DC1–CD8⁺ T-cell interactions and create an immunoregulatory niche influencing sensitivity to immune checkpoint blockade. Therapeutic strategies that restore DC activity, such as Flt3L and CD40 agonist–based approaches or cDC1-focused vaccination, can reprogram CD4⁺ T-cell polarization, enhancing helper T-cell support for cytotoxic immunity while limiting Treg expansion. Thus, CD4⁺ T-cell fate as a central determinant of immune responsiveness and therapeutic outcome in PDAC.^120^

#### Tregs

Tregs, a subset of either CD8+ or CD4^+^ T cells, contribute to immune suppression and create an environment that supports tumor progression.^121^ PDAC cells drive this immunosuppressive environment via tumor-intrinsic mechanisms and the secretion of several cytokines, such as TGF-β, IL-10, and IL-35.^122^ These conditions promote the upregulation and proliferation of effector Tregs, which hinder the anti-PDAC immune response.^122,123^ Since the discovery that increased Treg expression is a marker of poor prognosis in PDAC, Tregs have garnered wide attention as potential therapeutic targets for PDAC treatment.^124^ The role of Tregs in fostering an immunosuppressive milieu has been corroborated by other studies, highlighting their implication in PDAC progression and resistance to immunotherapy.^125^ Tregs are defined as CD4^+^ FOXP3^+^ CD25^+^ T cells. Activated Tregs express high CD39, TIGIT, and ICOS levels and can mediate immunosuppression. Sivakumar et al.^126^ found that Tregs were restricted to stromal and inflamed tissue and absent from epithelial sites. Furthermore, the majority of Tregs (90%) were in proximity to cytotoxic CD8^+^ T cells, thus facilitating their immunosuppressive action and dampening of cytotoxic T cell activity. These results show that the spatial dynamics within the PDAC-ME are conducive to immunosuppression and the hindrance of immune responses.

To uncover the role of distinct immune populations in PDAC progression, Sivakumar et al.^126^ conducted a comprehensive single cell multi-omics-based profiling study of lymphocytes isolated from the blood and tumors of 12 PDAC patients. They identified two distinct patient groups: an AE (adaptive immune cell)- enriched population and an ME (myeloid)- enriched population. The ME group was characterized by a worse prognosis and enhanced infiltration of myeloid subtypes, which were associated with immunosuppression and increased Tregs within the ME. These Tregs were highly activated and expressed immune checkpoints such as TGIT and CTLA-4. These findings suggest that ME patients may benefit from selective Treg targeting, such as anti-CCR8 immunotherapy.^127,128^ CCR8 has been recently identified as a marker of tumor-resident (Tr) Tregs, a “driver” of immune suppression.^129^ It is upregulated in nearly 80% of Tr Tregs following antigen presentation, but not on other immune cells, thus specifically defining this subtype. Depletion of CCR8-enriched Tregs using anti-CCR8 mAb has emerged as a potential approach for selectively targeting Tregs, inducing effective anti-tumor immunity and longer-lasting memory without eliciting immune complications, thus highlighting the importance of selective therapeutic strategies.^130,131^ Recent work by Suzuki et al.^126^ has shown that the ανβ5 integrin, which binds to proteinases and matrix macromolecules, is expressed only on tissue-infiltrating Tregs, defining a highly immunosuppressive CCR8^+^ Treg phenotype. This selective expression can be harnessed to target the cyclic peptide iRGD to PDAC-resident Tregs, thus specifically targeting activated Tregs. These studies highlight the heterogeneity of Treg populations within the PDAC-ME and the importance of understanding their roles and functions in the ME to develop efficient treatment approaches with minimal adverse effects.

**Fig. 3:**
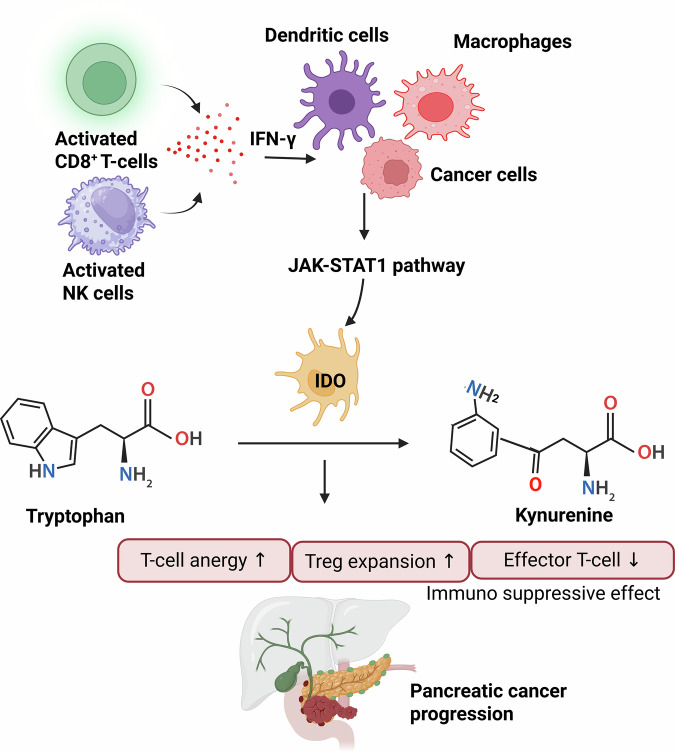
Indoleamine 2,3-dioxygenase (IDO)- mediated tryptophan catabolism drives immune evasion in pancreatic ductal adenocarcinoma (PDAC). IFN-γ produced by activated CD8⁺ T cells and NK cells induces IDO expression by tumor-associated antigen-presenting cells (APCs; dendritic cells, DCs, and macrophages). IDO plays a role in tryptophan catabolism, producing kynurenine, which inhibits effector T-cell proliferation and function while stimulating Treg activity. IDO also inhibits T-cell proliferation and function. By inhibiting anti-tumor immune responses, these immunoregulatory and metabolic changes contribute to PDAC progression. Created in BioRender.com

As previously mentioned, activated Tregs overexpress various ICMs, including glucocorticoid-stimulated TNFR-related protein (GITR), TIM-3, LAG-3, OX40, PD-1, and ICOS,^132^ further contributing to the creation of an immunosuppressive milieu in PDAC. These immune checkpoints include: (1) GITR, which modulates immune responses; (2) LAG-3, which inhibits effector T cells; (3) TIM-3, which negatively regulates T-cells (IFN-γ-secreting); (4) OX40, which controls T-cell activation; (5) PD-1, which suppresses it; and (6) ICOS, which influences T-cell co-stimulation.^132^ The complex interplay among these checkpoints highlights the heterogeneity of Treg populations and the complex pathways that mediate immune evasion in PDAC. Furthermore, the immune checkpoint CTLA-4 can bind to B7 receptors, thereby downregulating available B7 molecules (Fig. 2). CTLA-4 is upregulated in Tregs, which exhibit an elevated CD80/CD86 ratio on effector immune cells.^133^ This can competitively inhibit B7 binding to CD28 receptors, thereby inhibiting T cell activation and promoting immune suppression in PDAC.^134^ In response to inflammatory and immunoregulatory stimuli, the PDAC-ME induces IDO, which is not constitutively expressed at high levels. IDO catalyzes the conversion of tryptophan to kynurenine, leading to local tryptophan depletion and the accumulation of immunosuppressive metabolites (Fig. 3).^135^ By promoting T-cell anergy and Treg expansion while inhibiting effector T-cell proliferation and cytokine production, these metabolic changes aid immune evasion in PDAC (Fig. 3). Although PDAC cells themselves may express IDO under certain circumstances, tumor-associated APCs, such as DCs and macrophages, are the main source of IDO (Fig. 3).^136,137^ Interferon-γ (IFN-γ), produced by activated T cells and natural killer (NK) cells during anti-tumor immune responses, is a main inducer of IDO.^138^ Ironically, by increasing IDO in myeloid and tumor cells, this immune activation-associated cytokine creates a negative feedback loop that strengthens immune suppression.

Apart from IFN-γ, immunosuppressive cytokines such as IL-10 and TGF-β are secreted by Tregs and directly interact with other cells to induce IDO.^139^ IDO expression is further enhanced by immune checkpoint pathway engagement, such as CTLA-4-mediated signaling between Tregs and APCs, thereby creating a tolerogenic niche.^140,141^ Increased understanding of the diversity and heterogeneity of Tregs will thus inform the development of more efficient immune therapeutic strategies capable of targeting the immunosuppressive PDAC ME.

### T (CD8^+^) cells

T (CD8^+^) cells represent one of the most important constituents of the antitumor immune response against PDAC, recognizing and killing tumor cells, thus exerting an anti-tumor effect.^110^ However, the PDAC-ME can influence T cell cytotoxicity through the presentation of tumor Ags by APCs, including DCs. CD8^+^ T cells proliferate into effector T cells, then translocate into the ME to kill PDAC cells by releasing granzymes and perforin, which induce programmed cell death (apoptosis).^110^

Despite their important role in tumor patrol, several immune suppressive mechanisms reduce the function of T (CD8^+^) cells within the PDAC-ME. TGF-β, IL-10, and IDO can impair the function of T (CD8^+^) cells, thereby promoting immune evasion by PDAC cells.^142^ Furthermore, PD-1 or CTLA-4 expressions on the surface of T (CD8^+^) cells can impair these lymphocytes’ effector function upon ligation of their ligand on PDAC cells or immune cells, leading to T cell exhaustion and dysfunction. IL-17A-secreting T cells (CD8^+^) stimulate PDAC progression through stimulation of iCAFs.^143^ A recent study reported that a personalized mRNA–lipoplex neoantigen vaccine (autogene cevumeran) prompts de novo, high-avidity T cells CD8⁺ with long-term persistence and tissue-resident memory-like cytotoxic function in PDAC patients, and vaccine-induced T-cell responses are connected with significantly prolonged recurrence-free survival.^144,145^ Thus, stating that mRNA-based cancer vaccines can overcome durable T-cell priming barriers in PDAC.^144,145^ Xing et al.^146^ reported that B4GALT5 plays a crucial role in PDAC immune invasion by demonstrating that B4GALT5 elevated expression is associated with poor survival and impaired CD8⁺ T-cell cytotoxicity. Functionally, B4GALT5 promotes endoplasmic reticulum (ER) mediated degradation of MHC-I complex, which diminishes antigen presentation and enables tumors to escape CD8⁺ T-cell immune surveillance; thus, B4GALT5 is a promising immunotherapeutic target in PDAC.^146^ Thus, in PDAC, CD8⁺ T cells are significant mediators of antitumor response but are functionally suppressed by an immunosuppressive TME and tumor-intrinsic immune evasion process, while emerging strategies such as neoantigen mRNA vaccines and targeting B4GALT5 have the potential to restore durable cytotoxic T-cell responses and improve patient outcomes.

### NK cells

NK cells, a subtype of cytotoxic lymphocytes, are part of innate immunity and play an essential role in PDAC cell elimination.^147^ NK-cells sense a definite lack of MHC-I molecules by their killer-cell IgG-like receptors.^148^ Activated NK cells can produce different types of cytokines and chemokines, which stimulate and recruit adaptive and other innate immune cells.^149^ The function and activation of NK cells are suppressed by various mechanisms in the PDAC-ME. For instance, TGF-β secreted by PDAC cells can activate CAFs and generate an immunosuppressive environment, decreasing NK group 2D receptor (NKG2D), IFN-γ, granzyme B, and perforin, thus leading to PDAC resistance.^150,151^ CAFs can also secrete ILs and other signal molecules that impede the function of NK cells by inhibiting DNAM-1, NKG2D, IFN-γ, granzyme, and perforin.^150,152^ Furthermore, FAK in CAFs is inversely associated with NK cells’ function.^153^ The FAK/Src pathway is a key suppressor of NK cells’ activity.^154^ NK cell activity can also be inhibited by MDSCs via different mediators, TGFβ, adenosine, and IDO.^155,156^ Additionally, KRAS mutations can promote PDAC progression and metastasis by suppressing NK cell function^157^ whereas normal PTEN expression preserves NK cell function.^158^ The adhesive molecule CD155 is highly expressed in PDAC patients. It inhibits NK cell activity by decreasing CD226^+^ and CD96^+^ NK cells, thereby promoting immune escape.^159^ The enzyme nicotinamide phosphoribosyltransferase (NAMPT) is also involved in the inhibition of NK cells and resistance to chemotherapy in PDAC.^160^ Furthermore, DNA methylation can inhibit NK cell activity in PDAC.^161^ The overexpression of the transcription factor GATA6 induces NK cell activity by reducing stemness factors, SOX9, ALDH, and CD133.^151^ Hence, understanding the mechanisms of NK cell inhibition in the PDAC milieu, as well as tumor features and PDAC-ME changes, is vital for developing more efficient diagnostic tactics. It will also inform the expansion of NK cell-based therapies, ICIs, and the management of treatment responses, eventually improving PDAC-patient outcomes. As these distinctive characteristics reveal, NK cells represent promising targets for PDAC immunotherapy, corroborated by a number of clinical and preclinical investigations demonstrating their potential outcomes in various malignancies.

**Fig. 4:**
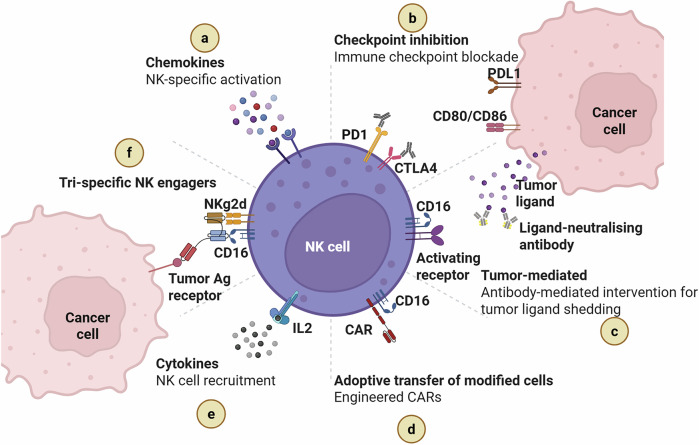
Approaches for NK cell immunotherapy. Different approaches activate NK cells, including the use of chemokines, checkpoint inhibition, ligand-neutralizing antibodies, recombinant CARS, cytokines, and Tri-specific NK cell engagers. An outline of the main strategies for enhancing NK cell activity against cancer cells. **a** NK cell activation and recruitment in the pancreatic tumor microenvironment are facilitated by chemokines. **b** NK cell suppression caused by tumor-expressed ligands (e.g., PD-L1, CD80/CD86) is alleviated by immune checkpoint inhibition, which includes blocking inhibitory receptors like PD-1 and CTLA-4. **c** Antibody-based tactics counteract tumor ligand shedding by enhancing NK cell cytotoxicity through ligand neutralization and antibody-dependent cellular cytotoxicity (ADCC) via CD16 engagement. **d** Targeted identification and destruction of tumor cells are enabled by the adoptive transfer of genetically engineered NK cells, such as those engineered with chimeric antigen receptors (CARs). **e** Cytokines like interleukin-2 (IL-2) support NK cell activation, proliferation, and survival. **f** To improve synapse formation and cytotoxic responses, tri-specific NK cell engagers concurrently connect tumor antigens, NK cell activating receptors (such as NKG2D), and CD16. When combined, these tactics seek to enhance NK cell-based cancer immunotherapy and combat tumor immune evasion. Created in BioRender.com

NK cell-based therapies offer a novel strategy to address the complexity of this malignancy (Fig. 4).^162^ Adoptive NK cell transfer applies to NK cells from patients or donors, followed by ex vivo expansion and activation to enhance their killing capabilities. The origin of NK cells has become an area of intense research, ranging from umbilical cord blood to peripheral blood and engineered cell lines. Genetic engineering has emerged as a powerful tool in NK cell therapy. CAR-NK cell technology introduces CARs into NK cells, allowing for precise targeting of cancer cells expressing specific Ags.^162^ This approach aims to overcome the challenges posed by immunosuppressive PDAC-ME.

Combinatorial strategies are at the forefront of research, with studies investigating the synergy between NK cell therapy and ICIs. ICIs aim to release the brakes on the immune environment, potentially improving the effectiveness of NK cell-mediated anti-cancer responses.^162^ Cytokines such as ILs can activate and sustain NK function. For example, IL-2 and IL-15 can increase NK cell anti-PDAC activity and have yielded promising results in preclinical and early clinical studies.^163^ As the field advances, researchers are uncovering the mechanisms of immune evasion employed by PDAC.^163^ Tailoring NK cell therapies that target the immunosuppressive milieu and unique antigenic landscape of pancreatic tumors is a focus of ongoing investigations. Clinical investigations are presently assessing the safety profile, efficacy, and long-term outcome of these innovative approaches to improve therapeutic interventions.

Ongoing trials are investigating several strategies, including NK cell therapy as monotherapy, genetic manipulation of CAR-NK cells, immunomodulator combinations, cytokine-induced stimulation methods, and allogeneic NK cell therapy. Adoptive NK cell therapy trials investigate the efficiency of autologous or allogeneic NK cell infusions, expanded ex vivo, in targeting and eliminating cancer cells. Genetic modification approaches engineered for NK cells, including CAR-NK cells, are being tested to enhance their specificity against cancer Ags (Fig. 4). Adoptive NK cell therapy, using autologous/allogeneic NK cells expanded ex vivo, is actively studied to improve PDAC targeting, with genetic engineering approaches, including CAR-NK cells, further improving antigen specificity.^164^ A recently developed NK cell specific PD-1–based chimeric switch receptor (PD1-CSR), incorporating DAP10, DAP12, and CD3ζ signaling domains, converts inhibitory PD-1 signaling into activation, with enhanced NK cell degranulation and cytokine production against autologous CD138⁺PD-L1⁺ malignant plasma cells in treated patients.^165^ Combination therapy trials aim to synergize NK cell therapy with ICIs to maximize anti-tumor responses. Cytokine stimulation trials explore the administration of ILs like IL-2 or −15 to enhance NK cell initiation and function. Allogeneic NK cell trials investigate the safety and efficiency of infusing NK cells from healthy donors into cancer patients.^166^ As the clinical landscape evolves, ongoing research seeks to refine NK cell therapies and improve treatment outcomes in PDAC.

## Immune checkpoint molecules (ICM)

ICMs play pivotal roles in regulating T-cell responses within the complex milieu of PDAC.^117^ Despite their fundamental roles in the immune system, these molecules operate through distinct mechanisms that affect immune responses and T-cell function. CTLA-4, found on the surface of T cells, and CD28 engage in a competitive interaction for binding to B7 ligands on antigen-presenting cells (APCs).^167^ By successfully outcompeting CD28, CTLA-4 disrupts the critical interaction between CD28 and B-7, thereby impeding the essential signal transmission required for T-cell activation.^167^ CTLA-4 effectively constrains the immune reactions of cytotoxic T cells directed towards PDAC. The strategic blockade of CTLA-4, achieved through anti-CTLA-4 antibodies like ipilimumab, holds promise in reinstating silent immune responses and augmenting the anti-tumor immune arsenal.

PD-1 is found in active lymphocytes and various immune cells. It directly hinders T-cell function by attaching to PD-L1 and 2. PD-L1, which is widely expressed on both immune and PDAC cells, establishes a positive feedback loop that hampers T-cell activation.^168^ This loop recruits the SHP2 tyrosine phosphatase, which dephosphorylates CD28, thereby weakening the TCR signal. The communication between PD-L1 and PD-1 can induce apoptosis, facilitating the tumor’s immune evasion. Independently, PD-L1 and PD-1 act as immunosuppressive molecules, collectively reducing the capacity of lymphocytes to infiltrate the ME.

### LAG-3

LAG-3 interacts with MHC receptors on APC membranes and plays a key role in immune responses to PDAC.^167^ MHC receptors bind to T-cell receptors (TCRs), actively participating in the initiation and T-cell proliferation. Notably, LAG-3 exhibits an increased affinity for MHC class II molecules, a vital component of the MHC system.^167^

MHC class II plays an essential role in immune recognition by presenting Ag’s to TCRs, thereby mediating T-cell activation.^169^ However, LAG-3 interferes with this process by blocking contact between MHC molecules and TCRs. This directly hinders the transduction of the TCR signal, thus indirectly impairing the immune response. LAG-3 exerts multifunctional effects on distinct subpopulations of immune cells in the context of PDAC.^170^ Its expression on CD4^+^ T cells brings about a regulatory dimension, weakening their functionality. LAG-3 expression on CD8^+^ T cells weakens cytotoxic functions and significantly impairs the immune system’s capability for targeting and eradicating PDAC effectively. The presence of LAG-3 on Tregs increases their immunosuppressive activity, enriching the environment with cytokines.^171^ Moreover, LAG-3 influences NK and B cells, indicating an additional role in the innate and adaptive resistance against PDAC. The engagement of LAG-3 with APCs has a negative effect on T-cell functions in the context of PDAC. The LAG-3 expression by a broad range of immune cell types and its capability for dampening active CD4^+^ T cells while promoting immunosuppressive functions underlines the relevance of LAG-3.

### TIM-3

TIM-3 expression is considerably elevated in PDAC tissue compared to healthy pancreatic tissue. It functions as an immune checkpoint within the ME,^172^ exerting its immunosuppressive effects through interactions with ligands on critical immune cells. Notably, co-expression of PD-1 and TIM-3 on TILs has been linked with poor clinical prognosis in PDAC patients.^173^ The role of TIM-3 in the context of PDAC is linked not only to its increased expression level but also to a network of interactions with various ligands, all of which contribute to its immunosuppressive role in the ME. Galectin-9, a major driver of the TIM-3-mediated immunosuppression, mediates the loss of CD4^+^ T cells, especially interferon-gamma-producing subsets, thereby resulting in an immunosuppressive ME within PDAC.^174^ This multi-faceted function of TIM-3 encompasses both direct and indirect immune regulation.

TIM-3 can identify non-protein ligands, such as phosphatidylserine, which are normally externalized on apoptotic cells, resulting in immune escape. Paradoxically, the interaction of TIM-3 with IFN-γ, otherwise known as a stimulator of immune responses, further complicates its function by modulating the activities of immune cells.^175^ These diverse pathways reflect the highly nuanced and multifaceted effects of TIM-3 on immune responses in PDAC and may represent new targetable strategies for therapeutic intervention.

### B7-H3

B7-H3 (CD276) plays a dual role in the PDAC-ME. Despite its putative positive function in T-cell initiation and IFN-γ secretion, B7-H3 has emerged as an inhibitory molecule that dampens T-cell activation.^64^ This inhibitory function is prominent as the B7-H3 ligand is upregulated on the surface of various activated NK cells, APCs, and T cells. Despite its broad and widespread expression, a definitive receptor for B7-H3 has not yet been known. The role of B7-H3 in inhibiting both NK and T cells highlights its regulatory function in PDAC immunomodulation. Elucidating the exact mechanisms by which B7-H3 mediates its inhibitory effects is essential for tailoring new B7-H3-based therapeutic strategies to modify the immune response in PDAC.

## TCR associated with immune checkpoints

**Fig. 5:**
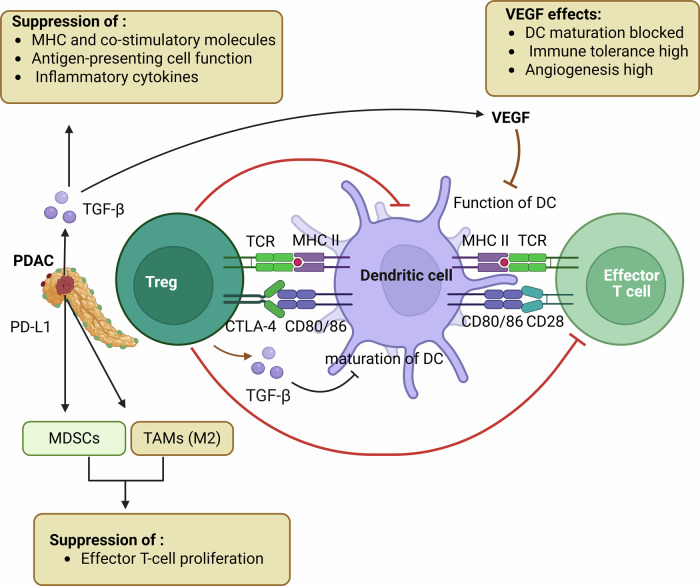
Tregs suppress the proliferation of dendritic cells (DC) and effector T cells. In PDAC tumor-derived TGF-β, VEGF, and PD-L1 overexpression suppress DC maturation. Tregs interact with dendritic cells through TCR with MHC II and CTLA-4 with CD80/86. Tregs interact with effector T cells through the TCR, as well as with MHC II and CD80/CD86, via CD28. TGF-β inhibits maturation, and VEGF inhibits the function of DC cells. MDSCs and M2-polarized TAMs reinforce the immunosuppressive environment. Created in BioRender.com

The correlation of peripheral T-cell receptor profiling with ICI responses in PDAC is an interesting area to explore and research. TCR profiling involves diversity and compositional analyses of the TCR repertoire in the ME.^176,177^ Increased understanding of the specific TCR repertoire in the periphery has helped predict and assess responses to ICIs, providing valuable insights. TCRs in peripheral blood may reflect the overall immune response to PDAC and the sensitivity of PDAC to ICIs (Fig. 5).^178^ Generally, a more diverse TCR repertoire is associated with a strong, responsive immune system, which could enhance ICI outcomes.^176,177^

The peripheral TCR profiles correlated with responses to ICIs, including antibodies such as anti-CTLA-4 and anti-PD-1 is an area of extensive research in PDAC (Fig. 5). Thus, TCR profiling, which measures the diversity and composition of TCRs in peripheral blood or ME, has emerged as a promising predictive biomarker of ICI efficacy. Integration of ipilimumab and nivolumab with peripheral TCR profiling points toward a comprehensive understanding of the immune environment in PDAC.^176,177^ Unique TCR signatures associated with positive responses to these ICIs may provide a rationale for personalized treatment approaches and further optimize immunotherapy based on each individual’s unique immune profile.

## Immunotherapy in PDAC

### Immunotherapy overview

**Fig. 6:**
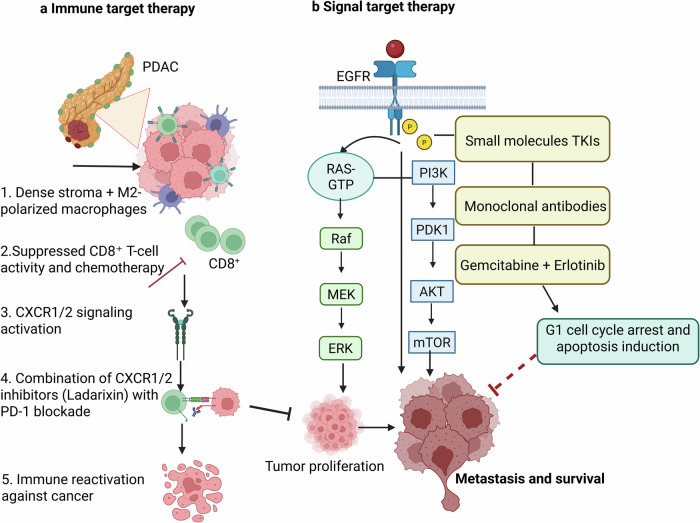
Targeted therapies in PDAC. **a** Immunosuppressive TME in PDAC limits chemotherapy’s action on tumor cells due to M2-polarized macrophages. While combined CXCR1/2 inhibition by ladarixin and PD-1 blockade restores antitumor immune reactivation as the inhibition of the CXCR1/2 pathway reduces immunosuppression, enhancing CD8⁺ T cell activation and exerting an antitumor effect. **b** Representation of EGFR signal transduction in PDAC in tumor progression. The growth ligand binds to the receptor, triggering a conformational change and autophosphorylation, which activate downstream growth signal cascades involving Ras-Raf-Mek and AKT-mTOR. Therapeutic interference by small-molecule tyrosine kinase inhibitors, monoclonal antibodies, and chemotherapeutic agents, as well as PD-1 blockade, such as gemcitabine plus erlotinib, inhibits these pathways, causing G1 cell cycle arrest and induction of apoptosis. Created in BioRender.com

In PDAC, immunotherapies have minimal efficacy due to a desmoplastic immunosuppressive environment that leads to an immunologically “cold.” But in combination with chemotherapy, radiotherapy, or ablative approaches that induce immunogenic cell death and disrupt immune evasion.^3,179^ Inhibition of CXCR1/2 with ladarixin modulates the ME in PDAC, as M2 macrophage polarization is reversed, reducing tumor burden and improving the efficacy of anti-PD-1 in resistant and humanized PDAC models (Fig. 6a). Thus, combining ladarixin with anti-PD-1 transforms PDAC from an immune-suppressive to an immune-permissive state.^180^ A phase II clinical trial (NCT03104439) assessed the combination of radiation, ipilimumab, and nivolumab in patients with metastatic microsatellite-stable CRC and PDAC who were resistant to immune checkpoint blockade. Among 40 CRC and 25 PDAC patients, the disease control rates were 25 and 20%, respectively, with improved outcomes. Pretreatment surgeries showed a low tumor mutational burden but enhanced NK cell and HERVK RNA expression; thus, combining radiation with immune checkpoint inhibitors may help overcome intrinsic resistance in MSS CRC and PDAC.^181^ Immunotherapy for PDAC is being actively explored in clinical trials.^75^ The current immunotherapy clinical trials, including those involving patients with PDAC, are summarized in Table 2.

**Table 2:** Immunotherapy-associated clinical trials in pancreatic cancer

Trial number	Status	Conditions	Intervention/Treatment	Ages eligible for study	Target	Trial phage	Study start to end date
NCT07153289	Not yet recruiting	Metastatic pancreatic cancer	CD318-CAR-T cells	≥18 years	CD318	Phase 1 Phase 2	2026-07
NCT07049055	Not yet recruiting	Metastatic pancreatic ductal carcinoma, pancreatic neoplasms	E-EDV-D682 (PNU-159682-packaged Nanocells) & EDV-GC (Glycolipid-Packaged Nanocells) + Gemcitabine Nab paclitaxel.	≥18 years	EGFR	Phase 1 Phase 2	2025-09
NCT06573398	Not yet recruiting	Resectable pancreatic ductal adenocarcinoma	SBRT with Sequential AG regimen + Tislelizumab + Thymalfasin	18 Years to 75 Years	T-cells, antigen-presenting immune cells, PD-1 receptor	Phase 2	2024-09-01
NCT05927142	Recruiting	Metastatic pancreatic cancer	Durvalumab & Rintatolimod combination therapy	≥18 years	PD-L1 and TLR-3.	Phase 1 Phase 2	2024-01-09 to–
NCT04157127	Recruiting	Pancreatic adenocarcinoma; Pancreatic cancer; Pancreatic adenosquamous carcinoma	Th-1 DC cell immunotherapy in combination with standard chemotherapy adj peg-IFN	≥18 years	Th-1 mediated activation of cytotoxic immune pathways	Phase 1 DECIST	2020-08-03
NCT06411691	Recruiting	Pancreatic and colon cancer	KRAS vaccine with Poly-ICLC adjuvant + Balstilimab + Botensilimab	≥18 years	Mutant KRAS	Phase 1b	2024-11-04
NCT06544655	Recruiting	Advanced solid tumors, including pancreatic cancer	BMS-986484 + Nivolumab + Oxaliplatin + Capecitabine Fluorouracil + Calcium folinate	≥18 years	CD40 and FAP	Phase 1	2024-10-10
NCT05013216	Recruiting	Pancreatic cancer High risk cancers	Mutant KRAS -targeted long peptide vaccine + Poly-ICLC	≥40 years	Mutant KRAS	Phase 1	2022-04-11
NCT06782932	Recruiting	Pancreatic cancer	Neoadjuvant and adjuvant GVAX vs a mutated KRAS peptide vaccine given with anti-PD-1 and anti-CD137	≥18 years	Mutant KRAS, PD-1 and CD137	Phase 1 Phase 2	2025-05-27
NCT05431270	Recruiting	Pancreatic ductal adenocarcinoma, Non small cell lung cancer	Mavrostobart (PT199) (Anti-CD73), Tislelizumab (Anti-PD-1), Gemcitabine + Nab-paclitaxel Docetaxel Pemetrexed Gemcitabine Carboplatin + Pemetrexed Pembrolizumab + Carboplatin + Pemetrexed	≥18 years	CD73 and PD-1/PD-L1	Phase 1 Phase 2	2022-08-11
NCT06941857	Recruiting	Metastatic pancreatic cancer	Oxaliplatin Irinotecan Folinic Acid 5-Fluorouracil NC410 Nivolumab Ipilimumab	≥18 years	Targeting collagen and the LAIR-1/collagen interaction and PD1 receptor or CTLA-4	Phase 2	2025-09-04
NCT06831136	Recruiting	Pancreatic ductal adenocarcinoma	Neoadjuvant chemotherapy (NAC) Immunotherapy (pembrolizumab) Endoscopic ultrasound (EUS)-guided radiofrequency ablation (RFA)	≥18 years	PD-1	Phase 2	2025-03-26
NCT06843551	Recruiting	Metastatic pancreatic ductal adenocarcinoma and metastatic pancreatic cancer	Stereotactic body radiation therapy. Botensilimab (anti-CTLA4) Balstilimab (anti-PD-1)	≥18 years	CTLA-4 and PD-1 receptors	Phase 2	2025-06-18
NCT04753879	Recruiting	Metastatic pancreatic ductal cancer	Nab-paclitaxel Gemcitabine Cisplatin Irinotecan Capecitabine Pembrolizumab Olaparib	≥18 years	PD-1 and PARP	Phase 2	2021-09-29
NCT05632328	Active, not recruiting	Pancreatic ductal adenocarcinoma pancreatic cancer Advanced pancreatic ductal adenocarcinoma	AGEN1423 (anti-CD73-TGFβ-Trap bifunctional antibody) and Botensilimab (anti-CTLA-4) w/ or w/o Chemo (Gemcitabine & Nab-paclitaxe)	≥18 years	CD73-TGFβ and CTLA-4	Phase 2	2024-08-08
NCT03727880	Active, not recruiting	Pancreatic ductal adenocarcinoma, Resectable pancreatic ductal adenocarcinoma	Pembrolizumab Defactinib	18 Years to 100 Years	PD-1, and FAK	Phase 2	2019-06-04
NCT04117087	Active, not recruiting	Pancreatic cancer, Colorectal cancer	KRAS peptide vaccine with poly-ICLC adjuvant + Nivolumab & Ipilimumab	≥18 years	Mutant KRAS, PD-1 and CTLA-4 receptors	Phase 1	2020-05-28
NCT03806309	Active, not recruiting	Pancreatic ductal adenocarcinoma	OSE2101 alone or in combination with nivolumab, followed by FOLFIRI	≥18 years	PD-1	Phase 1	2021-04-10
NCT06119217	Active, not recruiting	Pancreatic cancer	TTX-030, Nab-paclitaxel, and gemcitabine TTX-030, budigalimab, Nab-paclitaxel, and gemcitabine	≥18 years	CD39, PD-1 receptor	Phase 2	2024-03-25
NCT04999969	Active, not recruiting	Locally advanced or metastatic solid tumors	AZD0171 Durvalumab Gemcitabine Nab-Paclitaxel	18 Years to 130 Years	LIF, PD-L1	Phase 2	2021-12-10
NCT05726864	Active, not recruiting	Pancreatic cancer, Colorectal cancer	ELI-002 7 P immunotherapy	≥18 years	KRAS mutations (G12D, G12R, G12V, G12A, G12C, G12S, and G13D)	Phase 1/2	2023-04-14
NCT05945823	Active, not recruiting	Pancreatic cancer and other solid tumors.	Futibatinib Pembrolizumab Cisplatin 5-FU Oxaliplatin Leucovorin Levoleucovorin Irinoteca	≥18 years	FGFR1–4, PD-1	Phase 2	2023-07-13
NCT02451982	Active, not recruiting	Resectable pancreatic cancer	Cyclophosphamide GVAX vaccine Nivolumab Urelumab BMS-986253	≥18 years	DNA damage, immune cell activation, PD1 receptor, CD137 receptor, IL-8	Phase 2	2016-03-28
NCT05630183	Active, not recruiting	Metastatic pancreatic ductal adenocarcinoma	Botensilimab Gemcitabine Nab-paclitaxel	≥18 years	CTLA-4	Phase 2	2023-03-27
NCT00669734	Active, not recruiting	Pancreatic ductal adenocarcinoma, Pancreatic acinar cell carcinoma, Locally advanced pancreatic adenocarcinoma, Stage III pancreatic cancer AJCC v6 and v7, Stage IV pancreatic cancer AJCC v6 and v7.	Falimarev vaccine Inalimarev vaccine Sargramostim	≥18 years	CD80, CD54 and CD58 CEA and MUC-1 GM-CSF	Phase 1	2010-02-01
NCT04853017	Completed	Pancreatic ductal adenocarcinoma, Bile duct cancer, Cholangiocarcinoma and Colorectal cancer, etc.	ELI-002 2 P	≥18 years	Mutant K-RAS	Phase 1	2021-10-04 to 2024-09-24
NCT03193190	Completed	Pancreatic adenocarcinoma	Nab-paclitaxel Gemcitabine Oxaliplatin Leucovorin Fluorouracil Atezolizumab Cobimetinib PEGPH20 BL-8040 Selicrelumab Bevacizumab RO6874281 AB928 Tiragolumab Tocilizumab	≥18 years	PD-L1, MEK, hyaluronan (HA), CXCR4, CD40, VEGF, FAP, A2aR/A2bR, TIGIT, IL-6 receptor.	Phase 1/2	2017-07-05 to 2025-02-27
NCT03767582	Completed	Advanced pancreatic ductal adenocarcinomas	Stereotactic Body Radiation (SBRT) Nivolumab CCR2/CCR5 dual antagonist GVAX vaccine	≥18 years	PD1 receptor, CCR2 & CCR5, Delivering GM-CSF to the tumor site	Phase 1/2	2019-12-12 to 2024-09-30
NCT03250273	Completed	Pancreatic cancer Cholangiocarcinoma Metastatic pancreatic cancer Metastatic cholangiocarcinoma, etc.	Entinostat Nivolumab	≥18 years	HDAC1 and HDAC3, PD-1	Phase 1	2017-11-06 to 2020-11-20
NCT04336098	Completed	Advanced solid tumors	SRF617 Gemcitabine Albumin-bound paclitaxel Pembrolizumab	≥18 years	CD39	Phase 1	2020-03-16 to 2023-08-25
NCT04580485	Completed	Pancreatic ductal adenocarcinoma Bladder cancer Castration resistant Prostate cancer Colorectal cancer etc.	INCB106385 INCMGA00012	≥18 years	A2AR and A2BR PD-1	Phase 1	2021-02-03 to 2024-01-22
NCT03611556	Completed	Metastatic pancreatic adenocarcinoma	Oleclumab Durvalumab Gemcitabine Nab-paclitaxel Oxaliplatin Folinic acid 5-FU	18 Years to 101 Years	CD73, and PD-L1	Phase 1/2	2018-06-21 to 2022-07-22
NCT03549000	Terminated	Pancreatic ductal adenocarcinoma, Colorectal cancer Microsatellite stable (MSS), Metastatic castration resistant prostate cancer (mCRPC), Non-small cell lung cancer (NSCLC)	NZV930 PDR001 NIR178	≥18 years	CD73, PD-1 and A2A-R	Phase 1	2018-07-18 to 2022-10-17
NCT04989387	Terminated	Advanced solid tumors gastrointestinal (GI) malignancies, Squamous cell carcinoma of the head and neck (SCCHN)	INCA00186 Retifanlimab INCB106385	18 Years to 90 Years	CD73, PD-1, A2AR and A2BR	Phase 1	2021-10-04 to 2024-09-19
NCT04683939	Terminated	Pancreatic cancer, Biliary tract cancer, Cholangiocarcinomaand Esophageal adenocarcinoma etc.,	BNT141 mRNA-encoded anti-CLDN18.2 antibody, Nab-paclitaxel Gemcitabine	≥18 years	Claudin 18.2–expressing tumors	Phase 1/2	2022-01-18 to 2023-07-24

A multicenter Phase I clinical trial (NCT04806464) examined VG161, an oncolytic herpes simplex virus expressing IL-12, IL-15, IL-15Rα, and a PD-1/PD-L1-blocking fusion protein, in patients who had failed prior therapies. VG161 was tolerated, with no toxicities, and showed efficacy by selectively lysing tumor cells, releasing neoantigens, and reshaping the TME. As VG161 re-sensitizes resistant tumors, it stimulates systemic antioncogenic immunity. A gene expression–based efficacy prediction model identified patients most likely to benefit, and these patients correlated with improved overall survival.^182^ A phase II randomized, double-blinded, placebo-controlled clinical trial (NCT03331562) assessed pembrolizumab with or without the vitamin D receptor (VDR) agonist paricalcitol as maintenance therapy for PDAC. VDR agonists remodel the TME and sensitize PDAC to PD-1 blockade. PDAC patients with decent performance received pembrolizumab with either paricalcitol or placebo, but no benefit was seen in survival duration or toxicity. The lack of efficacy was attributed to paricalcitol’s short half-life, suggesting that longer-lasting VDR activation is needed to modulate the TME effectively.^183^ In a phase II trial (ChiCTR2000037927), SHR-1701, a bifunctional fusion protein targeting PD-L1 and TGF-β, in combination with famitinib, was studied in cases of biliary tract cancer or PDAC after failure of standard therapy. SHR-1701 plus famitinib demonstrated supporting efficacy in BTC patients. The regimen was well tolerated, with grade 3–4 TRAEs and no grade 5 events. Investigative analyses recognized immune metabolic signatures, immune phenotypic changes, and resection history as predictors of benefit, and an immune/metabolism score was proposed as a biomarker for patient selection. Thus, the approach simultaneously targets PD-L1, TGF-β, and angiogenesis pathways in refractory BTC and PDAC.^184^ The SAFFRON-104 trial (NCT03941873) evaluated sitravatinib, a tyrosine kinase inhibitor of TAM receptors TYRO3, AXL, MER, VEGFR-2, KIT, and MET, alone or in combination with tislelizumab, an anti-PD-1 antibody, in HCC. In phase I, I standardized the sitravatinib dose. In contrast, phase II assessed efficacy and safety. Alone, sitravatinib achieved a 25% response rate in pretreated HCC, whereas the combination with tislelizumab, an anti-PD-1 therapy, showed a modest response. Sitravatinib exerts antitumor effects by targeting pro-tumorigenic kinases that regulate angiogenesis, tumor growth, and immune evasion, while tislelizumab restores antitumor immunity through PD-1 blockade.^185^ In a phase II study, the combination of PD-1 inhibitor toripalimab and angio-immuno kinase inhibitor surufatinib in metastatic PD-L1–positive NSCLC and previously treated SCLC achieved 57.1% ORR. In disparity, the SCLC cohort showed a modest ORR of 15.8%.^186^ The PROSPER trial (DRKS00014054) investigated perioperative repurposing of propranolol, a non-selective β-blocker, and etodolac, a COX-2 inhibitor, in PDAC surgery to inflammation driven recurrence. The study was prematurely terminated due to slow recruitment, but safety outcomes were favorable.^187^ A multicohort transcriptomic analysis investigated that T-cell subpopulations reshape the TME and influence immunotherapy in PDAC. In different melanomas, there is a considerably higher infiltration of activated-potentially anti-tumor (APA) T-cells, low tissue-resident memory (TRM) T-cells, and reduced stem-like and dysfunctional T-cell subsets. In melanoma, elevated levels of stem-like TILs, TRM, dysfunctional T-cells, APA T-cells, and BTN3A were associated with improved response to inhibitors of PD-1/PD-L1 or CTLA-4. In contrast, ovarian and PDAC had fewer of these cells, with weaker responses to treatment. The study was conducted under the Total Cancer Care Protocol and Avatar® project (NCT03977402). Thus, PDAC remains largely resistant to immune checkpoint blockade; rational combinations with agents that remodel the TME, enhance T-cell activity, or disrupt immune evasion mechanisms could be effective strategies to transform PDAC into an immune-responsive disease.

### ICIs and chemotherapy or chemoradiotherapy

The combination of ICIs and chemotherapy is an interesting area of current investigation in PDAC. ICIs have shown limited success as single PDAC agents.^176,177^ However, researchers are exploring their efficacy in combination with chemotherapy to address these challenges.

Adding ipilimumab to gemcitabine did not yield additional benefits, as evidenced by comparable ORR.^188^ This demonstrates the challenge of determining the optimal combination regimens for PDAC, highlighting the need to improve clinical outcomes for this aggressive cancer. Recent work has shown that the combination of gemcitabine with tremelimumab was disappointing in chemotherapy-naive PDAC patients. Indeed, the study reported a median OS of 7.4 months 95% CI of 5.8–9.4, suggesting the at best marginal efficacy of this combination.^189^ A neoadjuvant regimen combining ICIs (anti-PD-1 IgG4 Ab) with capecitabine and radiation,^190^ significantly increased the median survival rate of patients, yielding positive outcomes. The combined use of immunotherapy, chemotherapy, and radiation may thus overcome the mechanisms of resistance observed in PDAC and enhance therapeutic efficiency, paving the way for multimodal and customized therapeutic strategies.

Despite major development in cancer immunotherapies such as ICB, CAR T-cell therapy, and cancer vaccines, PDAC remains highly refractory. Clinical trials using anti–PD-L1 and anti–CTLA-4 therapies, alone or in combination with chemotherapy, have failed to improve survival, due to intrinsic resistance mechanisms within the PDAC ME, which is dominated by immunosuppressive myeloid cells such as CCR2⁺,TAMs, CXCR2⁺,TANs, and mast cells.

### ICI and IDO

Navoximod (an IDO1 inhibitor) was combined with atezolizumab (an anti-PD-L1) in a Phase 1b safety and tolerability study.^191^ This combination is being studied in patients with advanced pretreated cancers to exploit possible synergies between IDO1 inhibition and ICI. Another Phase I/II study evaluated the safety and efficacy of durvalumab plus epacadostat in patients with metastatic solid tumors.^192^ The study found that the combination was well-tolerated but subtherapeutic, indicating that higher doses of epacadostat should be evaluated.

### ICI and mRNA-based vaccines

Despite the efforts to improve clinical outcomes in PDAC patients with combination strategies incorporating ICIs with targeted therapies or chemotherapy, clinically meaningful benefits have remained elusive. A phase I clinical trial evaluated the efficiency and safety of a new adjuvant treatment approach combining the autogene cevumeran, mRNA neoantigen vaccine with atezolizumab (anti-PD-L1) and modified FOLFIRINOX in resected PDAC patients.^145,193^ The combined therapy was well tolerated, as evidenced by this study. A significant finding from this study is the robust T-cell response elicited by autologous cerumen in 50% of the patients involved in the clinical trial.^194^ Increased T cell activity improved survival, resulting in a longer period of disease-free survival. The results highlight the potential of mRNA-based cancer vaccines for effective T-cell activation in an adjuvant setting, thus representing a promising strategy for improving outcomes in PDAC.^194^ While such results are promising, their transferability to a larger PDAC patient population warrants further research.

### Pairing adoptive T-cell therapy with an anti-PD-1

The combination of adoptive T-cell therapy and anti-PD-1 in PDAC represents a potentially revolutionary strategy.^195^ So far, immunotherapy, particularly anti-PD-1 antibodies, has yielded poor outcomes in PDAC due to the immunosuppressive PDAC-ME and low mutational burden.^178^ Adoptive T-cell therapy consists of collecting T cells from patients, altering them ex vivo to improve their anti-PDAC activity, and then reinfusing them into the patient.^196^ This approach aims to bolster the immune system’s capacity to specifically target and kill PDAC cells. However, T cells face many barriers within the PDAC-ME, including PD-1/PD-L1, which dampen T-cell function and promote PDAC immune evasion.^117^

Combining adoptive T-cell therapy with anti-PD-1 therapy addresses these challenges synergistically.^197^ Adoptive T-cell therapy provides a direct, targeted attack on PDAC cells, while anti-PD-1 therapy releases the brakes on T-cell activity, potentially enhancing their effectiveness within the immunosuppressive PDAC-ME.^197^ Preclinical studies and early clinical trials have shown favorable signs of efficacy, demonstrating improved T-cell infiltration into tumors, enhanced tumor cell killing, and, in some cases, durable patient responses. However, clinical translation in PDAC faces significant hurdles. The PDAC-ME is uniquely hostile, characterized by dense stroma, hypoxia, and Tregs and MDSCs.^198^ These factors can obstruct T-cell trafficking and function even in the presence of anti-PD-1 therapy, limiting the effectiveness of adoptive T-cell therapy combinations. Enhancing the invasion of T cells into tumors involves infusing a patient’s collected tumor-infiltrating lymphocytes (TIL).^199^ Despite PD-1’s well-established role in suppressing T cells, the Rosenberg group showed that PD-1 expression on TILs can identify the autologous, clonally expanded tumor-reactive lymphocyte repertoire, thus highlighting the dual role of PDL-1 in the PDAC-ME.^200^ The adoptive transfer of patient TILs is a multistep process that leverages a patient’s TILs to eradicate cancer cells. Patients first undergo a biopsy to collect tumor samples for autologous TILs expansions. Expanded TILs are infused back into patients by a non-myeloablative lymphocyte-draining treatment, incorporating chemotherapy and pembrolizumab. TIL infusion occurs with aldesleukin (IL-2 analog) and pembrolizumab.^201^ This approach suggests that combination therapies may be useful against PDAC, thus corroborating approaches that integrate vaccines and ICI treatments. A phase II trial is currently underway to explore the combination of ICI and adoptive T-cell therapy in PDAC patients.

Further investigation is required to optimize treatment protocols, identify predictive biomarkers of response, and address potential toxicities associated with heightened immune activation. Strategies to overcome immunosuppressive barriers, such as targeting additional immune checkpoints or combination with stromal-targeting agents, are actively being explored.

## Genetic and epigenetic alterations driving PDAC

### Chromosomal instability, altered copy numbers in PDAC progression

Previous studies on PDAC have been limited to identifying specific nucleotide mutations, overlooking the broader genomic changes.^202^ But in recent times, studies have revealed that chromosomal instability (CIN), validated as copy number alterations (CNAs) and structural reorganization, is a persistent driver of neoplasia transformation and metastatic progression.^203^ CIN and CNAs, as a key oncogene, enhance metastatic proficiency along with promoting tumor invasion through triggering cytosolic DNA sensing and immune signaling pathways, including STING-dependent mechanisms in PDAC.^204^ Also, CIN creates a genetically varied landscape that nurtures the evolution of prometastatic subclones.^204^ A comparative genomic analysis of primary and metastatic lesions showed that metastasis frequently arises from distinct subpopulations enriched in oncogenic CNAs.^205^ Synergistic interactions between point mutations and CNAs promote metastatic probability. For example, KRAS or MYC amplifications evidently intensify downstream signaling, whilst loss of function alterations in tumor suppressors like SMAD4 and TP53 promote genomic instability and invasive behavior.^206^ Thus, making metastatic progression in PDAC often dependent on intensifying prevailing oncogenic programs through CNAs and altered gene dosage. Along with genetic alterations, epigenetic mechanisms also shape the metastatic potential of PDAC. Aberrant chromatin remodeling, histone modifications, and DNA methylation vigorously reprogram the tumor transcriptomes to maintain invasion and dissemination.^207^ Reduced repressive H3K9 methylation and enhanced H3K27 acetylation activate the invasion-associated gene network system.^208^ Dissimilar to fixed mutations, histone modifications are reversible, allowing tumor cells to adapt transcriptionally to metastatic pressures. Loss of the histone demethylase KDM6A increases PDAC progression, while weakening p300 expression, which promotes metastasis.^209^ Analogously, transcription factor mediated reprogramming leads to chromatin remodeling; FOXA1 promotes anchorage-independent growth, and aberrant p63 expression determines the squamous transcriptional program linked to metastasis.^210^ Altered DNA methylation patterns further stabilize these invasive states, although direct fundamental links to metastasis require deeper understanding. However, the presence of diverse molecular subtypes in PDAC complicates the elucidation of subtype and metastasis associations.^211,212^ Yet, defining the subtype of metastatic lesions remains clinically significant, as basal-like and classical tumors display distinct therapeutic sensitivities.

### Molecular subtypes, oncogenic and therapy resistance driving pathways in PDAC

In a broader sense, the PDAC tumors can be classified into classical and basal-like subtypes, though some display hybrid features. These subtypes vary in oncogenic signaling and clinical outcomes. Predominant allelic imbalances favor mutant KRAS with high dissemination and are enriched in basal-like tumors, which also display activation of EMT, MYC, and cell cycle pathways, which are orchestrated by transcription factors like Snail, Slug, Twist, and ZEB1/2 and regulated by oncogenic KRAS-driven pathways, like TGF-β, Notch signaling, and Wnt/β-catenin.^213^ The MYC oncogene, a downstream of KRAS, controls metabolic reprogramming, cellular development, and biosynthetic processes by controlling genes involved in glycolysis, nucleotide synthesis, and ribosome biogenesis.^214^ MYC causes uncontrolled proliferation and collaborates with KRAS to propel tumor progression. Dysregulated cell cycle control in PDAC, driven by CDKN2A/p16 loss and TP53 mutations, permits unchecked proliferation under oncogenic stress and DNA damage, while integration with EMT and MYC triggers tumor aggressiveness and therapy resistance.^214,215^ Wide-range of exome sequencing studies reveal that the most recurrent KRAS alterations occur at codon 12 D, V, and R. In contrast, KRAS G12C is common in non-small cell lung cancer, and rare in PDAC; it evidently suppresses tumor development, even in the setting of TP53 loss,^216^ making it a central therapeutic target. Clinically, patients with basal-like tumors experience worse survival, parallel to those with classical subtypes. Subtype distribution also varies by metastatic site, with basal tumors affecting the liver and classical tumors more commonly associated with lung dissemination. These patterns correspond to differences in replication stress and immune responses. Although basal tumors are generally more aggressive, classical tumors are efficiently disseminated through circulating tumor cell clusters and epithelial-like states that facilitate colonization.^217^ PDAC tumors retain plasticity, with subtype switching occurring during progression, and primary tumors often contain mixed subpopulations, further complicating therapeutic targeting. Table 3 summarizes targetable molecular drivers of PDAC along with current clinical efforts against these targets.

**Table 3:** Targetable signals pathways and oncogenic drivers

Target	Drugs	Cancer Type	Phase	Clinical Trial Number
EGFR	Erlotinib in combination with gemcitabine	PDAC	First-generation TKI	–
EGFR	Cetuximab in combination with radiotherapy and gemcitabine	Progressive PDAC	Phase II	–
IGF-1R	MK0646 plus gemcitabine	PDAC	Phase I/II	–
EGFR / MEK / BCL-xL	Afatinib in combination with trametinib and DT2216	PDAC	–	–
EGFR / MMP-28	Erlotinib	PDAC	Preclinical	–
VEGFR	Ivonescimab (AK112)	PDAC	Phase I multicenter	NCT04597541
VEGF/PD-L1	Bevacizumab plus atezolizumab	Progressive PDAC	Phase II	NCT03074513
KRAS G12C	Sotorasib	PDAC	Phase I/II	NCT03600883
KRAS G12C	D3S-001	KRAS G12C mutant	Phase 1a/1b	NCT05410145
KRAS /MEK	Afatinib plus selumetinib	KRAS-mutant, PIK3CA WT PDAC	Phase I/II	–
Immunotherapy	Radiation plus ipilimumab and nivolumab	Metastatic PDAC	Phase II	NCT03104439
Immunotherapy	VG161 (oncolytic virus with IL-12/IL-15/PD-1 blocker)	PDAC	Phase I	NCT04806464
Immunotherapy	Pembrolizumab presence or absence of paricalcitol	PDAC	Phase II	NCT03331562
Anti-inflammatory	Propranolol + Etodolac	Perioperative PDAC	PROSPER trial	DRKS00014054
Immunotherapy	Total Cancer Care Protocol	PDAC (transcriptomic analysis)	Cohort study	NCT03977402

Mutations at codons 12, 13, and 61 in KRAS lead to constitutive formation of RAS–GTP and locking RAS in its active form to continuously send growth and survival signals, without activation of upstream signals controlled by growth factors or receptor tyrosine kinases (Fig. 6b).^216^ Early studies aimed at developing GTP-binding antagonists were unsuccessful. KRAS binds GTP with extremely high affinity, in the picomolar range, and its binding is irreversible. Furthermore, its structure lacks a pocket where a drug could fit and block the binding; thus, the initial drug development attempts failed.^218^ Alternate approaches were adapted to disrupt KRAS–effector interactions, inhibit the membrane localization, or target downstream pathways of RAF/MEK/ERK and PI3K/AKT/mTOR (Fig. 6b). Farnesyltransferase inhibitors (lonafarnib, tipifarnib) could advance to Phase III clinical trials but showed inadequate efficacy in KRAS-driven tumors.^219^ The first-generation RAF inhibitors approved for melanoma were ineffective in KRAS-mutant cancers, as they inadvertently activated CRAF and restored ERK signaling. Due to these breakdowns, focus shifted to target downstream effectors in the MAPK pathway, with the development of second-generation RAF inhibitors (LY3009120, PLX8394) and MEK inhibitors (trametinib, cobimetinib).^220,221^ Though in KRAS-mutant PDAC, these drugs were not much effective due to pathway “re-wiring” blocking RAF or MEK activated compensatory ERK reactivation and feedback loops that restored the signal and allowed PDAC cells to proliferate. Targeting MAPK and PI3K/AKT/mTOR pathways together showed promising results, but has the limitation of toxicity.^222^ Since KRAS necessitates plasma membrane association for its oncogenicity, post-translational modifications were targeted for therapeutic vulnerabilities. These modifications comprise farnesylation of the C-terminal CAAX motif, cleavage by RCE1, methylation by ICMT, and consequent palmitoylation or polylysine-mediated anchoring.^223^ Farnesyltransferase inhibitors again failed in clinical trials. Still, inhibitors of PDEδ, a prenyl-binding protein that facilitates KRAS trafficking to the plasma membrane, showed preclinical efficacy, albeit with concerns of off-target effects.^224^ KRAS also coordinates with metabolic reprogramming by enhancing nutrient acquisition through macropinocytosis and rewires glucose metabolism by upregulating transporters and GLUT1, HK_1/2_, PFK1, LDHA glycolytic enzymes.^225^

KRAS G12D sidetracks glycolytic intermediates into the hexosamine biosynthesis pathway, which supports protein glycosylation and the pentose phosphate pathway to generate ribose for nucleotide synthesis, mostly through MAPK and MYC signaling.^226,227^ MYC is indispensable for KRAS-driven metabolic gene expression and tumor maintenance. For years, KRAS was thought to be non-druggable, but recent developments in structure-based drug design have led to the authorization of KRAS G12C inhibitors (sotorasib, adagrasib) and the development of a KRAS G12D-specific inhibitor (MRTX1133), which has optimized PDAC treatment.^228^

Mutational loss of CDKN2A collaborates with KRAS stimulation and drives PDAC progression, yet CDK4/6 inhibitors alone show limited efficacy. A study demonstrates that combined inhibition of CDK4/6 and ERK-MAPK signaling synergistically suppresses PDAC aggressiveness by overcoming compensatory activation of ERK, PI3K, and MYC pathways. A CRISPR-Cas9 screen further identified multiple signaling nodes like CDK2, PI3K-AKT-mTOR, SRC kinases, and DNA repair pathways that augment CDK4/6 inhibitor sensitivity, while loss of RB1, PTEN, and FBXW7 conferred resistance.^229^ In a study, SWI/SNF-deficient pancreatic undifferentiated carcinomas, with SMARCB1, SMARCA2, or SMARCA4 loss, are strongly associated with rhabdoid or eccentric-nuclei morphology predominantly in younger patients, larger tumor size, and worse survival compared with SWI/SNF-retained tumors and conventional PDAC. These tumors also exhibited higher PD-L1 expression, with immune evasion potential. Prominently, a subset with SMARCB1 deletions appeared to be KRAS wild type, representing a distinct genomic profile with high aggressiveness and poor prognosis. SWI/SNF-deficient PDAC represents a unique molecular subgroup and benefits from novel therapeutic strategies, including EZH2 inhibition (NCT03213665), SMARCA2 degradation approaches (NCT05639751), and immune checkpoint inhibitors currently under evaluation in clinical trials.^230^ PDAC has a high risk of relapse even post curative surgery and neoadjuvant therapy (NAT) with good pathological response. This study evaluated KRAS mutations in tumor tissue, venous, and resection margin by digital droplet PCR. While NAT-treated tumors showed lower KRAS mutant allele frequencies (MAF) compared to up-front surgery (UFS) cases, and ypT1 tumors had higher MAFs than ypT0 remnants, KRAS status in margins did not predict recurrence-free or overall survival. KRAS-double negativity in venous and resection margins also lacked prognostic value. This study has a limitation of small cohort size; thus, further studies comparing margins from good versus poor NAT responders are needed to clarify the clinical utility of KRAS analysis.^231^ The COMPASS trial (NCT02750657) integrated whole genome and transcriptome sequencing in 268 advanced PDAC patients treated with either mFOLFIRINOX or gemcitabine-nab-paclitaxel, reporting median overall survival. The KRAS variants and allelic states alone lack prognostic value; however, basal-like PDAC with KRAS major imbalance (KRASmaj) associates with poor prognosis and higher prevalence of type II diabetes. HR deficient PDAC predicts better response to mFOLFIRINOX, while basal-like and systemic inflammation associated PDAC cohorts define aggressive disease, linked to altered immune profiles.^232^ The CCTG PA.7 trial (NCT02879318), a randomized phase II mPDAC trial, paralleled gemcitabine/nab-paclitaxel with or without durvalumab and tremelimumab. Combination immunotherapy did not improve (*p* = 0.72) overall survival and only showed mild toxicity. Though exploratory ctDNA analysis disclosed improved survival in patients with KRAS wildtype tumors across both treatment arms, highlighting the prognostic value of ctDNA-based KRAS mutation status in PDAC.^233^ A case study highlighted that although co-occurrence of KRAS and EGFR mutations in PDAC is extremely rare, treatment with gemcitabine plus the EGFR inhibitor erlotinib achieved a biochemical response and 7 months of disease control after progression on standard regimens, suggesting potential clinical benefit of EGFR-targeted therapy in such dual-mutant PDAC cases (Fig. 6b).^234^ In adenosquamous carcinoma of the pancreas (ASCP), a rare and aggressive variant of pancreatic PDAC, there is no recognized standard therapy. A report states that a 68-year-old male with metastatic ASCP with KRAS G12C mutation went through multiple systemic therapies, including KRAS G12C–targeted inhibition, an outstanding response to pembrolizumab, a single-agent immune checkpoint inhibition (ICI) despite intact mismatch repair proteins. While KRAS G12C inhibitors showed only modest benefit, pembrolizumab achieved significant disease control, suggesting that the distinct tumor microenvironment of ASCP, characterized by squamous differentiation, may contribute to enhanced immunogenicity and responsiveness to ICI.^235^ Pancreatic acinar cell carcinoma (PACC) report in an 81-year-old man who progressed on standard chemotherapy and had SEL1L-NTRK1 fusion. Initiation of targeted therapy with the NTRK inhibitor larotrectinib led to an exceptional and durable radiographic response, with disease control maintained for 13 months and no grade 3 toxicities.^236^ This first reported case of NTRK fusion in PACC underscores the importance of molecular profiling in rare pancreatic cancers. A prospective study of 378 PDAC patients revealed that while alterations were more common in KRAS wild-type tumors (31.1% vs 13.2% in KRAS-mutated), clinically relevant targets were still present across both groups, including BRCA1/2 mutations (7.5%) and a rare NTRK fusion. KRAS wild-type patients showed longer median overall survival (19.35 vs 16.89 months). Importantly, 22.3% of patients were deemed eligible for early-phase clinical trials, with some receiving matched therapies. Highlighting the significance of molecular profiling in PDAC, irrespective of KRAS status.^237^ This case describes a 74-year-old woman with KRAS G12D-mutated PDAC and multiple liver metastases who responded to systemic chemotherapy, enabling transition to an oligometastatic state. Subsequent multimodal metastasis-directed therapy (MDT): MWA for liver lesions and SBRT for the primary tumor achieved complete radiologic resolution and 20-month progression-free survival, despite the aggressiveness of KRAS G12D PDAC.^238^ Thus, combining MDT with chemotherapy could improve outcomes in selected patients.

### DNA damage repair defects as a therapy target in PDAC

DNA damage events in a cell are repaired by DNA repair mechanisms such as base and nucleotide excision repair, direct repair, mismatch repair, and double-strand break repair via homologous recombination (HR) or non-homologous end joining (NHEJ), and by checkpoint molecules to maintain genomic stability.^239^ Defects in DNA damage repair (DDR) leads to oncogenic modifications, aggressiveness, and resistance to therapeutics. DNA repair mechanisms comprise. Whilst HR provides precise repair using sister chromatids, NHEJ is error-prone and may introduce genomic instability. Germline mutations in HR genes, including RAD51C/D, CHK2, BRCA1/2, ATM, ATR, FANC, and PALB2, are linked to inherited PDAC, pointing towards DDR as a critical therapeutic target.^240,241^ Therefore, understanding how mutations disrupt DDR and checkpoint regulation gives opportunities for targeted therapies aimed at using defective repair machinery in PDAC.

The sensors and mediators of DNA repair are called Poly (ADP-ribose) polymerase (PARP) proteins, PARP1/2 particularly, for single-strand breaks (SSBs).^242,243^ During DNA damage, PARP senses the break and catalyzes the addition of ADP-ribose units to itself and other proteins by the PARylation process, which recruits other repair machinery.^244^ As PARP activity depends on cofactor nicotinamide adenine dinucleotide (NAD), this target can be used for PARP inhibitors (PARPi), which imitate NAD and trap PARP at sites of DNA damage, averting repair and with cytotoxic DNA lesions.^245^ This is most common in oncogenic cells with mutated genes of BRCA1, BRCA2 or PALB2 i.e HR repair-deficient cells.^246^ Clinical trials with one PDAC patient with an HR-related mutation, Olaparib and Rucaparib, a PARPi, showed improved response and progression-free survival.^247^ Despite the promise of PARPi in PDAC, its efficacy is limited in PDAC patients without HR defects. Moreover, combining PARPi with chemotherapy or other targeted therapies may improve anti-tumor effects but often increases toxicity. Furthermore, HR-deficient tumors may develop resistance via restoration of HR function, PARP mutations, or activation of alternative DNA repair pathways.^248^ It is vital to enhance the therapeutic potential of PARP inhibitors in PDAC.

Upon detection of DDR, specific molecules recruit downstream transducers to initiate signal cascades of the repair mechanism. Kinases like ATM, ATR, and DNA-PKcs control cell cycle checkpoints and DNA repair. ATM mutations are associated with pancreatic, breast, and prostate cancers, and silencing ATM increases oncogenic cell sensitivity to DDR inhibitors (DDRi), chemotherapy, and radiotherapy.^249^ The DSB repair machinery, on sensing DNA breaks, is initiated by the MRN complex (MRE11/RAD50/NBS1), which assembles repair proteins, stimulates checkpoints, and promotes repair.^250,251^ Oncogenic KRAS mutations create replication and transcription conflicts (TRCs), if unmitigated, cause DNA breaks and genome instability. However, the BER pathway regulates TRCs by modifying RNA Pol II and R-loop dynamics, thereby preventing excessive DNA damage.^252^ However, inhibiting BER increases ATR-Chk1 initiation, suggesting that dual inhibition of BER and ATR pathways may synergistically enhance oncogenic cell death.^253^ Thus, KRAS mutations initiate TRC events, supporting combined KRAS- and DDR-targeted therapy in PDAC. FAM110C and BEND4 genes are implicated in DDR regulation, where epigenetic silencing of BEND4 increases oncogenic aggressiveness in PDAC, while its knockdown increases sensitivity to ATM inhibitors.^254,255^ Thus, epigenetic therapies with demethylation agents are used to restore DDR activity and improve treatment outcomes.

Some patients with PDAC show BRCA1/2 and PALB2 mutations, which are associated with high tumor mutation burden (TMB) and mismatch repair (MMR) augmentation, which enhances response to immune checkpoint blockade (ICB) therapy. Such patients may also respond well to PARP inhibitors, particularly when combined with immunotherapy. Other DDR proteins influence therapy resistance. For example, DDB2 is overexpressed in radiation-treated PDAC, enhancing survival by activating cell cycle checkpoints and PARP.^256^ Thus, DDB2 could serve as a biomarker for predicting responses to radiation therapy. Goodwin et al.^229^ reported that the loss of CDKN2A disrupts the tumor-suppressor function of p16INK4A, which further synergizes with the KRAS mutation, increasing the aggressiveness of PDAC. Additionally, CDK4/6 inhibition shows limited efficacy alone. Combination of a CDK4/6 inhibitor (palbociclib) with an ERK-MAPK inhibitor (ulixertinib) synergistically suppressed PDAC proliferation by blocking compensatory activation of ERK, PI3K, anti-apoptotic signaling, and MYC. Phase I clinical trial (NCT03454035) was initiated to evaluate this combination in advanced PDAC patients. A CRISPR-Cas9 loss-of-function screen identified additional genes and pathways (CDK2, PI3K-AKT-mTOR, SRC kinases, HDACs, autophagy, chromosomal regulation, and DNA repair) whose inhibition augmented CDK4/6i sensitivity, while loss of RB1, PTEN, and FBXW7 conferred resistance. Thus, therapeutic combinations with CDK4/6 inhibitors may overcome resistance and improve outcomes in PDAC.^229^ Two parallel Phase II nonrandomized trials (NCT02677038) evaluated olaparib monotherapy in 46 patients with advanced PDAC exhibiting the “BRCAness” phenotype defined by non-germline-BRCA DDR alterations or ATM protein loss. The treatment was well-tolerated but demonstrated limited anti-oncogenic activity; median progression-free survival was 3.7 months, significantly longer in the DDR-GA subgroup (5.7 months, *P* = 0.008), and median overall survival reached 13.6 months in this cohort. Thus, olaparib may benefit a subset of platinum-sensitive PDAC patients harboring DDR genetic alterations, even in the absence of germline BRCA mutations.^257^ The DAPPER (NCT03851614) phase II study evaluated durvalumab combined with olaparib or cediranib in patients with mismatch repair pMMR-CRC and PDAC. In 31 pMMR-CRC and 19 PDAC patients, both combinations showed limited anti oncogenic effect, with few patients attaining stable disease or unconfirmed partial responses, and the majority showing disease progression. Treatments were generally well tolerated, with no grade 4–5 treatment-related adverse events. Baseline ME features, which have higher tumor-infiltrating lymphocytes and lower CD^68+^ cells, and distinct immune gene expression profiles, were associated with clinical outcomes. Thus, immune contexture may influence response in these immunotherapy-resistant cancers.^258^ PDACs show poor response to immunotherapy because of immunosuppressive TME, except in microsatellite instability-high (MSI-H) or mismatch repair-deficient (dMMR) cases. A case of a 45-year-old woman with Lynch syndrome and MSI-H/dMMR PDAC who received neoadjuvant pembrolizumab showed marked metabolic tumor regression, allowing successful R0 pancreatoduodenectomy. Postoperative histology showed substantial tumor reduction, increased CD^8+^ T cell infiltration, and enhanced anti-tumor immune response.^259^ To evaluate the safety and efficacy of the PARP inhibitor veliparib combined with FOLFOX chemotherapy in PDAC, in a phase I/II study, the recommended dose of veliparib was 200 mg twice daily in phase II. Among all patients, the overall objective response rate (ORR) was 26%, with higher activity observed in platinum-naive patients and with HR-DDR mutations, with an ORR of 57%. Thus, integrating veliparib with platinum-based therapy in this genetically defined subset of PDAC.^260^ In PDAC, less than 2% of patients show microsatellite instability, and these are likely to respond well to immunotherapy with immune checkpoint inhibitors.^261^

### Epigenetic targets in PDAC

PDAC can be driven by canonical mutations in KRAS, TP53, CDKN2A, and SMAD4, and can be promoted by genetic heterogeneity, with ~10% of patients showing germline DNA repair defects in BRCA1/2, PALB2, and ATM that promote genomic instability. Alternative subgroup displays mutations in epigenetic regulators comprising DNMT3A, TET2, SWI/SNF complex, and ASXL1, which disrupt chromatin arrangement and gene expression, thereby propelling malignancy.^262^ ARID1A, a tumor suppressor in PDAC, with mutation ~6% or loss triggers early precursor alterations like acinar-to-ductal metaplasia, PanINs, and cystic lesions; these alone are not enough for complete PDAC transformation. Additional oncogenic events, such as p53 inactivation or Myc overexpression, are necessary to overcome the growth constraints caused by ARID1A loss.^263^ SWI/SNF components comprising ARID1B, SMARCB1, PBRM1, SMARCA2, and SMARCA4/BRG1 are also mutated; notably, BRG1 loss cooperates with KRAS mutations to generate IPMN-like neoplasms, which is context-dependent, while it inhibits KRAS-driven PanIN formation in acinar cells, it promotes preneoplastic lesions in ductal cells, a cell type–specific chromatin remodeling in PDAC development.^264^ Transcriptomic profiling identified that frequent mutations in histone-modifying enzymes such as KDM6A (MLL1) and MLL2/3/4, which are present in all PDACs, synergize with KRAS stimulation to increase PDAC progression.^265^ Epigenetic deregulations drive genomic structural changes, forming a distinct PDAC transcriptional subtypes that span a continuum of phenotypes, extending from early lesions to aggressive forms.^213^ Thus, in PDAC, driver mutations remain conserved, but metastasis is propelled by large-scale epigenetic reprogramming that reshapes chromatin, heightens malignancy, and adapts tumors to new microenvironments.

Altered activity of HATs, HDACs, HMTs, and HDMs rewrite histone marks and chromatin states. Loss of repressive marks such as H3K9me2 and H3K27me3 in LOCK domains drives metastasis, while CDK9 inhibition remodels chromatin and restores chemosensitivity.^266^ To maintain stemness and immune evasion, a stroma-derived metabolite 2HG disturbs histone methylation. EZH2 and BMI1 initiate PDAC to a stem-like phenotype, therapy resistant, while KDM6A acts as a restraint on EZH2; its loss eliminates this restraint, remodeling enhancers and opening the opportunity for targeted therapy (e.g., BET inhibitors).^265,267^ p300/CBP facilitated super-enhancers strengthen oncogenic pathways through ΔNp63, MYC, RUNX3, and ZEB1 push squamous/basal identity. Subtype plasticity arises from epigenetic modifications in classical tumors that retain PDX1/GATA6 and KRAS dependence, whereas basal-like tumors lose KDM6A and 5hmC, gain EZH2 repression, and adopt aggressiveness and resistance to therapy.^268^ Thus, EZH2, BMI1, KDM6A, CDK9, BET proteins, p300/CBP and super-enhancers are at therapeutic vulnerability in PDAC. The noncoding RNAs comprising mRNAs, eRNAs, lncRNAs, siRNAs, circRNAs, and piRNAs, control transcription, post-transcriptional modification, and chromatin remodeling in PDAC, with some having oncogenic properties and others having tumor suppressor properties.^269^ Oncogenic mRNAs include mR-21;mR-27a;^270,271^ mR-146a;^272^ mR-196a;^273^ mR-200a, mR-155;mR-200;^274^ and mR-221/222^275^ propel PDAC dissemination and drug resistance, while tumor-suppressive mR-20a;^276^ mR-142-3p;^277^ and mR-34^278^ inhibit PDAC progression. Likewise, lncRNAs such as HOTAIR, MALAT1, NEAT1, and NUTF2P3-001 promote tumor progression, invasion, and PRC2-dependent repression, while GAS5 functions as a tumor-suppressive switch in CD133^+^.^279^ MeCP2 interacts with lncRNAs (MALAT1, NEAT1) to strengthen oncogenic process.^280^ Thus, deregulated ncRNAs are epigenetic regulators of PDAC initiation, progression, and therapy resistance.

## Emerging biomarkers for early detection and prognosis

### Short interfering RNA (siRNA)

**Fig. 7:**
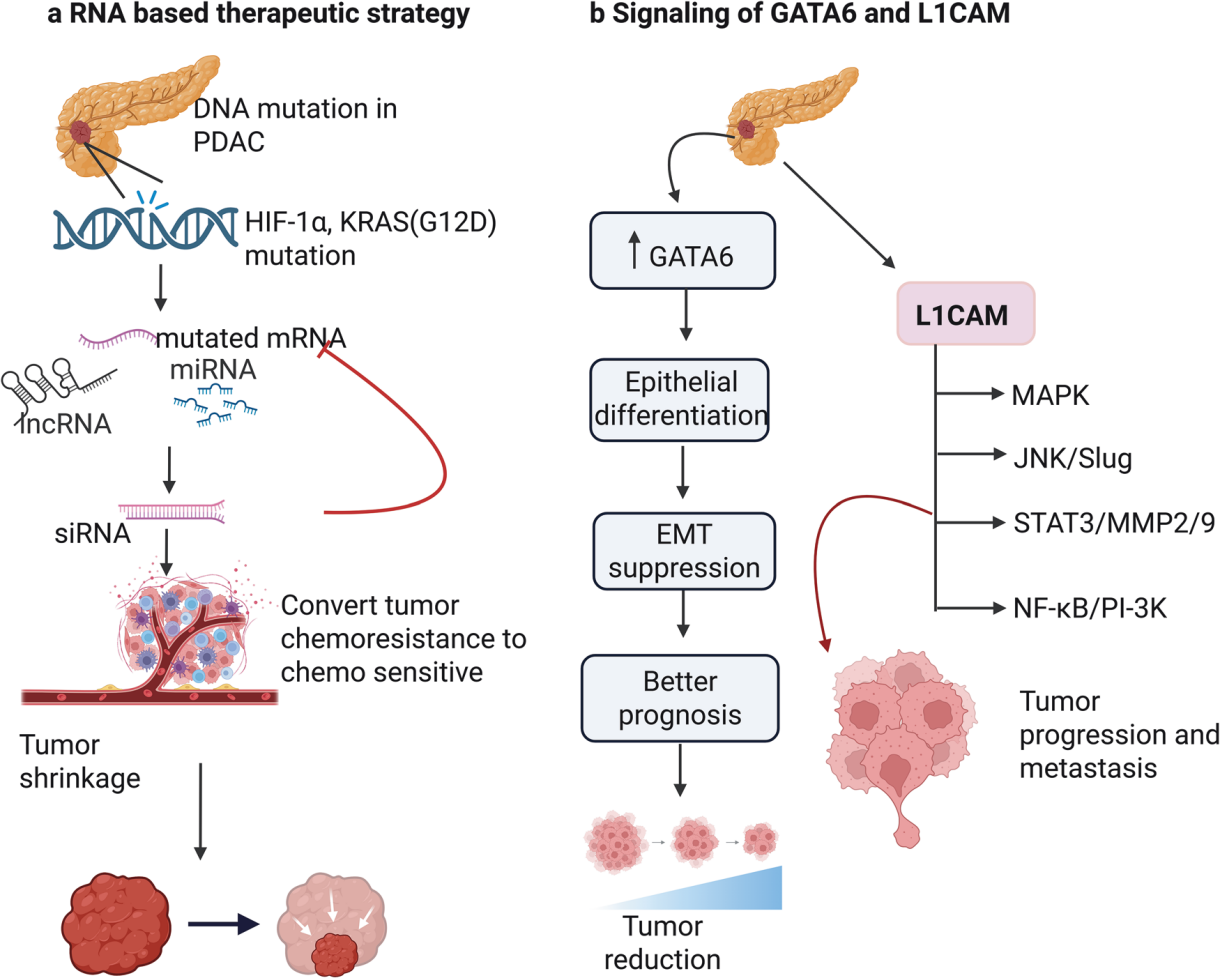
RNA-based and GATA6–L1CAM signaling as therapeutic strategies in PDAC. **a** siRNAs based therapeutics by targeting mRNA, lcRNA regulating as HIF-1α, KRAS(G12D) signals and overcome chemoresistance. lncRNAs, for example, DNAH17-AS1, dysregulate microRNA function, acting as oncogenes (mR-21, mR-155, mR-196a) or tumor suppressors (mR-34a, mR-145), which control tumor growth, survival, drug resistance, and dissemination during therapy, thereby converting to a chemosensitive environment. **b** GATA6 and L1CAM in PDAC progression and therapy response. Elevated GATA6 promotes epithelial differentiation, suppresses EMT, enhances MHC-I expression, increases CD^8⁺^ T-cell infiltration, and improves prognosis and chemotherapy response to FOLFIRINOX. L1CAM, produced by Schwann cells or TGF-β1, activates MAPK, JNK/Slug, STAT3/MMP2/9, NF-κB/PI3K signal, enhances EMT, metastasis, drug resistance, and immune evasion. Created in BioRender.com

siRNAs are short RNA molecules that silence target mRNAs and prevent protein translation without introducing new mutations. In PDAC, siRNA targeting HIF-1α has been shown to decrease mRNA and protein levels, thereby helping overcome chemotherapy resistance and inhibiting cancer progression, as discussed in Fig. 7a.^281^ A study focused on long noncoding RNA DNAH17-AS1, upregulated in PDAC, is interrelated with poor prognosis and survival. Silencing DNAH17-AS1 suppressed cancer cells’ sustainability and dissemination and promoted apoptosis. Mechanistically, DNAH17-AS1 acted as a competing endogenous RNA by sponging miR-432-5p, thereby preventing miR-432-5p from downregulating its direct target protein, which has a role in tumor progression called PPME1. From this, it is evident that the DNAH17-AS1/miR-432-5p/PPME1 axis is important in PDAC progression and could be a novel therapeutic target. To date, no specific drug targeting DNAH17-AS1 has been reported in clinical development; RNA interference-based therapies or antisense oligonucleotides targeting DNAH17-AS1 could represent promising strategies.^282^ A revealed that Forkhead box O6 (FOXO6) is overexpressed in HCC and correlates with tumor size, TNM stage, AFP levels, HBsAg status, and level of differentiation, thus serving as a prognostic biomarker. Mechanistically, FOXO6 silenced by siRNA decreased cell proliferation, promoted apoptosis, induced G0/G1 cell cycle arrest, increased p27, and reduced cyclin D1 expression. Mechanistically, FOXO6 promoted tumor progression by associating with oxidative stress, as HCC tissues with high FOXO6 showed elevated ROS, HO-1, SOD, GPx, and CAT. A search for specific drugs targeting FOXO6 is under investigation. N-acetylcysteine or HO-1 inhibitors, inhibitors of oxidative stress regulators, and small molecules targeting FOXO-related signaling may have translational prospects.^283^ Further, CD36, a multi-ligand scavenger receptor, is highly expressed in PDAC and is associated with venous microinvasion and poor prognosis. Patients with elevated CD36 expression exhibited reduced overall survival and recurrence-free survival; thus, CD36 is a prognostic factor in patients with gemcitabine adjuvant chemotherapy. CD36 expression was upregulated in gemcitabine-resistant PDAC, and its silencing via siRNA enhanced gemcitabine sensitivity, diminished anti-apoptotic proteins, restored apoptosis, and decreased chemoresistance.^284^ The siG12D-LODER™ implant, a miniature biodegradable device releasing siRNA against KRAS(G12D) and evaluated in Phase I/IIa trial (NCT01188785) for patients PDAC. Patients received intensifying doses of gemcitabine or modified FOLFIRINOX; the treatment was tolerated, and no tumor progression, stable disease, or two partial responses with CA19-9 reduction were observed.^285^ miR-29b-3p promoter methylation in PDAC: pyrosequencing and qPCR showed that hypermethylation of the miR-29b promoter reduced miR-29b-3p expression while upregulating DNMT1. High promoter methylation was observed in Bxpc3 and Capan-2 cells, correlating with low miR-29b-3p levels. Silencing DNMT1 increased tight junction proteins (ZO-1, occludin) and reduced cancer progression and angiogenesis, whereas miR-29b restored the inhibitory effects lost with DNMT1 overexpression.^286^ Thus, RNA interference is a promising strategy for overcoming chemoresistance and improving therapeutic outcomes in PDAC (Fig. 7a).

### microRNAs(mR)

mRs regulate oncogenic progression, metastasis, and drug resistance, and have emerged as potential biomarkers in PDAC, with diagnostic, prognostic, and therapeutic attributes due to their stability and specificity.^287^ mRs control gene expression by binding to the 3′ untranslated regions (3′UTR) of target mRNAs, degrading or repressing translation, thereby controlling proliferation, apoptosis, angiogenesis, and chemoresistance.^288^ Circulating mRs remain stable in blood, either bound to Argonaute 2 (Ago2) or encapsulated in extracellular vesicles, ultimately providing them with resistance to ribonuclease degradation and ideal candidates for noninvasive biomarkers.^289^ Research has identified diagnostic groups of circulating mRs that enhance sensitivity and specificity when combined with CA19-9, the conventional PDAC biomarker. For instance, panels incorporating mR-21, mR-210, and mR-181c demonstrated near 100% diagnostic accuracy when evaluated alongside CA19-9, while analysis at a large scale identified signatures of dysregulated mRNAs, as miR-21, as the most reliable biomarker for PDAC diagnosis and prognosis.^290^ Unique signatures, identified as early detection markers, are mR-1469, mR-4530, and mR-125a-3p.^291^ Prognostically, elevated levels of oncogenic mR-21 and mR-196a indicate poor survival and therapy resistance, while raised levels of tumor suppressor mR-34a and mR-451a improve treatment response.^292^ Mechanistically, mRs related to chemoresistance, as mR-21 and mR-200, modify gemcitabine sensitivity, while mR-34 re-establishes setbacks of drug resistance.^293^ Furthermore, certain mRs, like mR-218, mR-10a and mR-10b are connected with PDAC aggressiveness.^294^ A meta-analysis of 1525 PDAC patients showed that high mR-21 strongly predicts poor overall survival and disease-free survival, with additional associations for high mR-155, mR-203, mR-222, mR-10b, and low mR-34a.^270,271^ Another study demonstrated that mR-196a is upregulated in cancers and serves as both a diagnostic biomarker (AUC ~ 0.87, high sensitivity and specificity) and a poor prognostic marker. Higher expression in tumor or plasma correlates with reduced survival in PDAC.^273^ EV-derived mR-200, a plasma biomarker, is a diagnostic marker with an AUC of 0.97; sensitivity 100%, specificity 88% in PDAC, but not in benign pancreaticobiliary disease.miR-221 (258 mR) regulates multiple malignancies and inflammatory conditions, underscoring its role as a biomarker and therapeutic target, with miR-221 silencing warranting clinical investigation.^275^ ATAD2, an oncogenic driver in PDAC is targeted by hsa-mR-217, which binds its 3′UTR and suppresses its expression. Overexpression of mR-217 diminishes PDAC progression and induces apoptosis, arrests the cell cycle, and deactivates the progressive AKT signal pathway.^295^ A study identified that miR-146a-5p is impaired in PDAC and its loss promotes gemcitabine resistance through activation of the TRAF6/NF-κB/p65/P-gp axis; while reviving miR-146a-5p suppresses proliferation, enhances chemosensitivity (Fig. 7a).^272^ Thus, miRNAs are versatile biomarkers that not only aid in early diagnosis and prognosis of PDAC but also represent potential therapeutic targets for reversing chemoresistance.

### GATA6

GATA6, a member of a small family of zinc finger transcription factors, plays a vital role in regulating cellular differentiation and organogenesis and has emerged as a prognostic biomarker in PDAC, with elevated expression correlated with improved overall survival (Fig. 7b).^296^ In contrast, low levels of GATA6 expression correlate with bigger tumor size, metastasis, poor differentiation, and aggressiveness. GATA6 expression also affects chemotherapy response, with FOLFIRINOX, as patients with high GATA6 expression have better response and survival outcomes. In contrast, low GATA6 expression is associated with poor aggressiveness, as shown in Fig. 7b.^297^ In the basal-like PDAC subtype, loss of GATA6 causes poor differentiation and leads to resistance to therapy, while its high expression in the classical PDAC phenotype is linked to better prognosis.^298^ Mechanistically, loss of GATA6 drives a basal transcriptional program through ΔNp63^298^ and requires concurrent loss of HNF1A/HNF4A; it also controls tumor plasticity and immune evasion, as GATA6 deficiency decreases MHC class I expression and CD^8+^ T-cell infiltration.^298^ Also, GATA6 overexpression suppresses EMT markers. ESPAC-3, ESPAC-4, and the COMPASS trial have confirmed that low GATA6 expression predicts poor OS and chemotherapy resistance, independent of SMAD4 mutation status.^299^ Thus, GATA6 is a prognostic marker in PDAC.

### L1CAM

L1 cell adhesion molecule is expressed at elevated levels in PDAC and promotes aggressiveness and drug resistance in PDAC. Schwann cells release L1CAM and PDAC through the MAPK pathway, while stimulation of STAT3 upregulates MMP-2/9, promoting invasion (Fig. 7b).^300,301^ TGF-β1 produces its expression via the JNK/Slug pathway, independent of Smad proteins, causing EMT and drug resistance.^302^ In PDAC progression, L1CAM expression increases in tumor epithelium, relating to advanced grade, involvement of lymph node dissemination, and poor survival. Functionally, L1CAM recruits regulatory T cells and suppresses effector T cells, generating an immunosuppressive microenvironment that supports immune escape and tumor growth.^303^ Both full-length L1CAM (L1CAM-FL) and its soluble/cleaved forms sustain oncogenic signaling via integrins, NF-κB/PI3K/Akt; ERK; and FGFR pathways, improving tumor cell existence, proliferation, and stem-like characteristics, including self-renewal, resistance to drugs, dormancy, and EMT, as discussed in Fig. 6b. Elevated L1CAM expression is an independent prognostic factor for poor outcome and therapy resistance, including resistance to oxaliplatin.^301^ Thus, L1CAM emerges as both a diagnostic and prognostic biomarker in PDAC.

## Targetable signal pathways and oncogenic drivers

### Targeting transmembrane receptor proteins

The seven transmembrane G-protein coupled receptors (GPCRs) regulate varied biological processes, and their dysregulation is seen in tumor progression and dissemination, thus making them an efficient target. Glycoprotein epidermal growth factor receptor belonging to the ErbB family of tyrosine kinases, EGFR/HER1; HER2/3/4 dimerizes when ligand is bound to the receptor, causing autophosphorylation, and activation of RAS/MAPK and PI3K/AKT, downstream signaling molecules which promote tumor proliferation, persistence, and dissemination (Fig. 6b).^304^ EGFR is a therapeutic target due to its oncogenic role in PDAC. FDA-approved EGFR inhibitor, erlotinib (with gemcitabine),^305^ moderately enhanced the survival proportion but has been limited by toxicity and drug resistance.^306^ Monoclonal antibody inhibitors, such as a chimeric cetuximab Mab, bind to the EGFR and block ligand binding, which decreases the downstream signal transduction leading to the G1 cell cycle arrest and activates apoptosis.^307^ In the PARC pancreatic cancer treated with radiotherapy and cetuximab in a phase II trial, trimodal therapy (IMRT, gemcitabine, cetuximab) in locally advanced pancreatic cancer was safe and improved, but cetuximab did not significantly enhance overall survival compared to gemcitabine alone.^308^ In RAS wild-type metastatic colorectal cancer (mCRC), a single-arm phase II trial assessed switching from cetuximab to FOLFIRI plus bevacizumab based on early tumor shrinkage. Among 29 patients, 22 achieved ETS and continued cetuximab before switching, while 7 ETS-negative patients switched earlier. The switching reduced toxicities, and paronychia improved after the transition. Thus, maintaining mitigating toxicity when switching from anti-EGFR to anti-VEGF therapy.^309^ In a phase III trial comparing postoperative modified FOLFOX6 versus perioperative modified FOLFOX6 plus cetuximab in patients with KRAS wild-type resectable colorectal liver metastases, no significant difference in survival was observed; thus, adding perioperative cetuximab to FOLFOX6 did not improve outcomes.^310^ Many TKIs are used as first-generation in clinical practice, like erlotinib, alectinib, and gefitinib. The sage and tolerable regime includes gemcitabine, erlotinib, capecitabine, and oxaliplatin, which enabled surgical resection in some borderline resectable PDAC patients.^311^ Together, targeting EGFR through small-molecule inhibitors, monoclonal antibodies, and combination regimens demonstrates clinical potential in PDAC and other GI malignancies. However, efficacy is often limited by toxicity.

### Insulin like growth factor 1 receptor (IGF-1R)

A transmembrane protein, IGF-1R, is activated by the binding of the ligand insulin growth factor 1 and is associated with poor prediction. Preclinical studies have shown that dual targeting of EGFR and IGFR may affect PDAC progression and apoptosis through crosstalk in downstream signal transduction.^312^ A randomized Phase I and II clinical trial on gemcitabine in combination with IGF-1R antagonist (MK0646), compared to gemcitabine in combination with erlotinib with or without MK0646, demonstrated better survival for MK0646 combined with gemcitabine but no change in progression-free survival.^313^ The triplet schedule of MEK inhibitor (trametinib), BCL-xL degrader (DT2216), and pan-EGFR inhibitor (afatinib) showed cytotoxicity and tumor subdual compared to MEK/BCL-xL doublet therapy in PDAC models by targeting PI3K/AKT and p38 MAPK. Thus, EGFR blockade improved therapeutic response.^314^ Enhanced MMP-28 leads to increased TGF-α maturation, which activates the EGFR signal and promotes PDAC progression. PDAC with elevated MMP-28 is sensitive to erlotinib, as MMP-28, as a prognostic biomarker, can help identify responsive patients with advanced erlotinib’s efficacy at reduced dose and toxicity.^315^ Thus, further studies are required for better efficacy.

### Vascular endothelial growth factor receptor (VEGFR)

VEGFR is a tyrosine kinase domain of a transmembrane protein, with a ligand that activates angiogenesis signal transduction to the blood supply at tumor growth; overexpression of VEGF results in poor prognosis, and phosphorylation results in angiogenesis.^316^ Immunoglobulins such as ramucizumab and bevacizumab target the VEGF/VEGFR2 pathway, are FDA-approved for cancer treatment, but are not effective in PDAC. In a phase I multicenter trial (NCT04597541), ivonescimab (AK112), a first bispecific antibody targeting PD-1 and VEGF for PDAC, showed promising results, with sustained > 80% PD-1 occupancy and effective VEGF suppression.^317^ In a randomized Phase II trial, patients with advanced pNETs received everolimus ± bevacizumab with octreotide. Survival was better with a combination treatment involving VEGF pathway inhibitors.^318^ In a Phase II trial (NCT02795858), patients with advanced extra-pancreatic neuroendocrine tumors were treated with ramucirumab, a VEGFR-2 inhibitor, in combination with somatostatin analog. The regimen was safe and provided a window for further studies on VEGF/angiogenesis inhibitors.^319^ In a phase II study (NCT03074513), patients with advanced tumors were treated with bevacizumab a VEGF inhibitor plus atezolizumab a PD-L1 inhibitor and the combination led to responses in 20% PDAC with a progression-free survival.^320^ Thus, multiple studies focus on the potential of combining VEGF/VEGFR inhibition with immunotherapy or targeted agents to improve outcomes in PDAC warranting further clinical investigation.

### Ras signaling as a target

PDAC has frequent KRAS mutations, which drive neoplasia and dissemination. Heritably modified models have advanced understanding of KRAS-driven PDAC and emphasize the imperative need for effective RAS-targeted therapies (Fig. 6b).^321^ In a preclinical analysis, it was demonstrated that the combination of the HDAC inhibitor MPT0E028 with a MEK inhibitor produced synergistic reduction of cell survival in both KRAS-mutant and wild-type PDAC cells, with augmented apoptosis compared to each drug alone. Mechanistically, low doses for long-term therapy with MPT0E028 or HDAC inhibitors alone absurdly activate ERK and EGFR signal transduction, which limits efficacy. However, co-administration with a MEK inhibitor abolished constitutive ERK activation and strengthened apoptotic signals. Overexpression of HDAC4/6 or MEK reversed the cytotoxic effect, substantiating pathway dependence. In the AsPC-1 xenograft mice model, the combination therapy achieved a greater reduction in tumor volume compared to each single agent.^322^ The trial (NCT03600883) evaluated sotorasib, a first-generation covalent inhibitor of the KRAS p.G12C mutation in Phase 1 and II analysis. PDAC patients with advanced metastasis who had undergone treatment for KRAS p.G12C received sotorasib. Which selectively binds to the cysteine residue created by the KRAS G12C mutation in its inactive GDP-bound form, thus preventing activation of the downstream RAS/RAF/MEK/ERK proliferative signaling pathway.^323^ The CCTG PA.7 trial (NCT02879318) examined gemcitabine + nab-paclitaxel with or without durvalumab and tremelimumab in advanced PDAC. The combination immunotherapy failed to improve survival over chemotherapy alone (*p* = 0.72), though toxicity was manageable. Exploratory ctDNA analysis showed that patients with KRAS wildtype tumors had significantly better survival across both treatment arms.^233^ The Phase 1a/1b trial of D3S-001 (NCT05410145) evaluated this next-generation KRAS-G12C inhibitor in patients with metastatic KRAS-G12C mutations, in NSCLC, CRC, and PDAC. Mechanistically, D3S-001 was intended to improve target engagement and overcome growth factor induced nucleotide exchange, a known resistance mechanism to earlier KRAS-G12C inhibitors. In the study, D3S-001 showed dose-dependent pharmacokinetics, no dose-limiting toxicities, and the maximum tolerated dose was not reached efficacy was encouraging in the G12Ci-naive cohort, with 66.7% in NSCLC, 88.9% in colorectal cancer, and 75% in PDAC. In contrast, in G12Ci-pretreated NSCLC, the ORR was lower at 30%, though the disease control rate was 80%.^324^ A Phase I trial evaluated the combination of afatinib, a pan-HER inhibitor, and selumetinib, a MEK inhibitor, in patients with KRAS-mutant, PIK3CA wild-type tumors, including CRC, NSCLC, and PDAC. The rationale was that MEK inhibitors alone show limited efficacy in KRAS-driven oncogenesis due to feedback activation of EGFR signaling, which reactivates downstream MAPK and PI3K-AKT pathways. In combination with afatinib and selumetinib, the trial aimed to block this compensatory signaling and enhance antitumor effects. The safety and dose-limiting toxicities were observed. In Phase II, afatinib in combination with selumetinib showed moderate efficacy.^325^ Both the SBRT in combination with pembrolizumab and trametinib trial and the CCTG PA.7 trial (NCT02879318) discuss the challenges and opportunities of immunotherapy-based strategies in PDAC. In the SBRT established trial, the integration of CPI checkpoint inhibitor pembrolizumab in combination with radiotherapy and MEK inhibitor was associated with better survival benefit, as PD-L1 expression and immune cell infiltration (TILs) were linked to improved outcomes. In contrast, PD-L1 alone without TILs was less favorable. By comparison, the CCTG PA.7 trial, which combined gemcitabine/nab-paclitaxel with durvalumab (anti–PD-L1) and tremelimumab (anti–CTLA-4), failed to improve overall survival in an unselected PDAC population, and has limitations of immunotherapy without biomarker-driven stratification. Mechanistically, the SBRT in combination with pembrolizumab and trametinib approach leverages tumor antigen release by SBRT, immune checkpoint blockade, and MEK inhibition to overcome KRAS-driven resistance. In contrast, the PA.7 regimen relied on broad checkpoint inhibition without directly addressing KRAS or TME-driven immunosuppression.^326^ These investigations emphasize that targeting KRAS signaling and EGFR/MAPK pathways, and integrating biomarker-driven immunotherapy, are promising yet challenging strategies in PDAC.

### PARP and HDAC as targets

PARP inhibitors block protein ADP-ribosylation, impairing DNA repair, while HDAC inhibitors alter protein acetylation, affecting cancer cell genomic stability.^327^ The therapeutic activity of hydrogen sulfide (H₂S) donors in PDAC may be understood through the interplay with epigenetic and DNA repair pathways. At high concentrations, exogenous H₂S exhibits antioncogenic effects through oxidative stress, DNA damage, and apoptosis; however, at low concentrations, H₂S promotes cancer.^328^ Mechanistically, H₂S has been shown to inhibit HDACs, causing increased histone acetylation and transcriptional reactivation of tumor-suppressor genes, though decreasing BRCA1 and RAD51 protein, a homologous recombination repair. Thus, impairs DNA double-strand break repair. In parallel, H₂S-induced oxidative stress activates PARP, which resolves accruing DNA lesions through base excision repair. However, when HR repair is suppressed by HDAC inhibition, PARP-mediated repair alone becomes insufficient, resulting in a synthetic lethality-like state. Thus, the antioncogenic role of H₂S donors can be mechanistically linked to the HDAC–PARP axis.^329^ The therapeutic potential of combining CM03, a naphthalene diimide derivative that stabilizes G-quadruplex (G4) DNA structures, with the histone deacetylase (HDAC) inhibitor SAHA (suberanilohydroxamic acid) in PDAC, with gemcitabine-resistant models, was investigated.^330^ There was a synergistic effect at concentrations close to or below the individual GI50 values of each drug. Mechanistically, SAHA relaxes compressed chromatin by increasing histone acetylation, thereby exposing G-rich sequences and facilitating greater G4 formation. CM03 then stabilizes these G4 structures, leading to transcriptional repression of oncogenes, torsional strain on DNA, and accumulation of DSBs.^330^ The γ-H2AX marker and cleaved PARP indicated apoptosis.^330^ A novel HDAC inhibitor, CG200745, used to overcome gemcitabine resistance in PDAC, in combination with CG200745, gemcitabine and erlotinib, was evaluated in both gemcitabine-sensitive and resistant PDAC. CG200745 induced apoptosis by increasing the expression of cleaved PARP and caspase-3, while also raising acetylated histone H3 levels, due to HDAC inhibition. When combined with gemcitabine/erlotinib, CG200745 produced synergistic growth inhibition with ~50% reduction in tumor size. CG200745 sensitized gemcitabine-resistant cells by downregulating ATP-binding cassette (ABC) transporter genes, MRP3 and MRP4, responsible for multidrug resistance. HDAC inhibition relaxes chromatin, enhances drug availability, suppresses drug efflux, and induces apoptosis, thereby restoring gemcitabine efficacy. This preclinical study suggests that CG200745 holds promise as an effective chemosensitizer in PDAC.^331^ To overcome erlotinib resistance in PDAC by HDAC6-selective inhibitor ACY-241, it was demonstrated that hyperacetylation of its substrate α-tubulin reduced the viability of erlotinib-resistant PDAC. A synergistic effect was observed when ACY-241 was combined with erlotinib, leading to enhanced apoptosis via PARP cleavage and autophagy induction, which suppressed the AKT-mTOR signal while activating phospho-AMPK, thereby shifting cells toward autophagy-dependent death.^332^ Inhibition of siLC3B and siATG5 autophagy genes protected cells, confirming autophagy’s central role.^332^ A HDAC inhibitors TSA and VPA synergize with the PARP inhibitor AZD2461 to enhance DNA damage and reduce PDAC proliferation and survival, by downregulation of CHK1 and RAD51 via disruption of mutp53–HSP70 crosstalk and partial reactivation of wtp53.^333^ Combining panobinostat or vorinostat a HDAC inhibitors with talazoparib or olaparib, PARP inhibitors causes synergistic cytotoxicity in PDAC. The addition of decitabine further enhanced apoptosis and DNA damage, while impairing DNA repair, ATM, BRCA1, and ATRX, and altering epigenetic regulation.^334^ RUNX2/mutant p53/TAp63 regulatory axis determines the sensitivity of p53-mutated PDAC to SAHA. PDAC has poor response to SAHA alone, silencing mutant p53 or RUNX2 enhanced SAHA-mediated apoptosis, marked by increased γH2AX and cleaved PARP, along with upregulation of TAp63.^335^ Thus, targeting the HDAC–PARP axis, either alone or in rational combinations with H₂S donors, G4 stabilizers, radiosensitizers, or chemotherapeutic agents, represents a promising strategy to overcome drug resistance and improve therapeutic efficacy in PDAC.

## Therapeutic resistance challenges and mechanisms

Resistance to therapy is a major concern in PDAC, which contributes to poor prognosis. PDAC remains extremely resistant due to both innate and acquired mechanisms. Innate resistance factors include altered KRAS, TP53, SMAD4, and CDKN2A, which created a complex mutational landscape of PDAC, remodeling signaling networks, and the TME to favor progression and survival.^336^ Acquired resistance develops during treatment, with tumor cells evolving through selection, TME interactions, or metabolic rewiring. MAPK, PI3K/AKT/mTOR, and NF-κB control proliferation, immune evasion, and survival, while their crosstalk promotes compensatory signal transduction that undermines targeted inhibition.^337^ Overexpression of molecules like Neuropilin-1, LAMC2, and MUC1, MUC4 additionally drives resistance by triggering drug efflux transporters, hindering drug uptake, promoting EMT, and enhancing invasion. Further, Hedgehog signal promotes desmoplasia and stromal remodeling, limiting vascular perfusion and drug delivery.^338^ Resistance to therapy in PDAC involves multiple factors that contribute to poor prognosis, including immune evasion, metabolic rewiring, impaired drug delivery, and enhanced invasion.

### Molecular alterations

PDAC progression and therapy resistance are driven by primary mutations in KRAS, TP53, SMAD4, and CDKN2A, which rewire the signaling networks that drive PDAC aggressiveness.^262^ Mutant KRAS activates PI3K/AKT, MAPK, and NF-κB cascades, creating an environment for growth, cytokine production, and cross-pathway signaling that enhances tumor resistance. TP53 loss-of-function mutations reinforce chemoresistance by activating JAK2/STAT3 and upregulating cytidine deaminase to degrade gemcitabine, and by transforming the desmoplastic stroma by triggering pancreatic stellate cells, thereby preventing drug delivery.^254,339^ MAPK/ERK signaling promotes gemcitabine and 5-FU resistance while supporting PD-L1 expression to maintain immune evasion; however, MAPK inhibition can trigger compensatory PI3K/AKT activation.^340^ Constitutive active PI3K/AKT/mTOR promote tumor growth and BAD inactivation, while also supporting NF-κB activity, which drives inflammation, stromal transformation, and immune exclusion via CXCL12-mediated CD^8+^ T cell dominance.^341^ NF-κB and AKT signal confer resistance to gemcitabine, with AKT2 inhibition shown to restore chemosensitivity through PUMA.^342^ PDAC cells employ drug efflux mechanisms via MUC1- and LAMC2-facilitated upregulation of ABC transporters, impairing intracellular drug retention. MUC1 forms a mucin barrier that blocks uptake and activates Cox-2–dependent resistance, while MUC1/TGF-β crosstalk drives EMT and metastasis.^343^ MUC4 also stimulates drug resistance by disrupting gemcitabine transporters (hENT1, hCNT1/3), triggering HER2/ERK and NF-κB signal, and hindering apoptosis. The Hedgehog pathway orchestrates stromal desmoplasia by driving PSC and ECM deposition, thereby hindering perfusion and drug delivery. Impairing Hedgehog signaling reduces matrix density and restores gemcitabine sensitivity.^344^ Thus, these linked oncogenic mutations and compensatory signaling networks make PDAC highly adaptive to drug resistance.

### PDAC-ME-driven resistance

PDAC-ME is rich in PSCs and CAFs, which secrete growth factors to promote PDAC propagation, stimulate ECM, fibrosis, and stromal transformation, creating a dense, fibrotic, and hypoxic barrier that supports tumor development, metastasis, restricts blood flow and drug diffusion, creating a chemo-resistant environment.^17^ Hypoxia induced by hypovascularity stabilizes HIF-1α, promotes autophagy, metabolic adaptation, and gemcitabine-induced stemness through AKT/Notch1 signal cascade.^17^ As autophagy is a survival mechanism under nutrient stress, with PSCs supplying metabolites such as alanine to sustain PDAC growth.^17^ Triggered PSCs, driven by cytokines and growth factors TNFα, IL-6, TGF-β, and PDGF; Hedgehog signal, deposit extreme collagens, fibronectin, laminins, and ECM proteins, making resistance to chemotherapy via pathways like ERK1/2 and PI3K/AKT. Immune evasion is achieved through the secretion of Galectin-1, and control is exerted via the Notch signal.^17,345^ CAFs alter the ECM by secreting exosomes, which promote drug resistance through mTOR, NF-κB, and IL-1β–IRAK4 pathways.^346,347^ Stromal communications and hypoxia support CSCs, drive PDAC plasticity, immune evasion, and drug resistance via PI3K/ Wnt/ Hippo/ and Notch pathways.^345^ Exosomes mediate drug resistance by regulating MDR proteins, ncRNAs, metabolic rewiring, and immune suppression.^346,348^ Immune cells, regulatory T cells, TAMs, TANs, and MDSCs, create immunosuppression, ME, promote fibrosis, and reduce chemotherapy response through TGF-β1 and ADP/ATP signal.^348^ Thus, this complex network of stromal, immune, and tumor interactions creates a hostile and drug-resistant TME, making PDAC one of the most challenging cancers to treat.

### Altered tumor metabolism

The hallmarks of PDAC include altered metabolism to survive in a hypoxic and nutrient-deficient ME and resist therapy. The metabolic rewiring is achieved through the actions of KRAS, TP53, MYC, PTEN, and GNAS, which increase glycolysis, glutamine utilization, fatty acid oxidation, and pathways for scavenging, including autophagy and macropinocytosis.^349^ Crosstalk within stromal cells further fuels these adaptations, as PSCs and CAFs supply metabolites, such as alanine, lipids, lactate, and pyrimidines. Hypoxia stabilizes HIF-1α, promotes glycolysis, elevates lactate production, causing acidosis and EMT, which dampens T-cell function.^350^

Overexpression of MUC1 and MUC4 integrates metabolic reprogramming with activation of multidrug resistance genes and HIF-1α signaling, leading to impaired gemcitabine uptake and survival of resistant clones.^351^ CSCs exhibit exclusive metabolic states, often OXPHOS-dependent, yet plastic, contributing to therapy resistance and metastatic heterogeneity. Signal regulators such as AMPK and PI3K/AKT/mTOR act as metabolic sensors, modifying autophagy, redox balance, and survival, with their dual tumor-promoting and suppressive roles.^352^ Autophagy, driven by AMPK and stromal signaling, serves as a key adaptive mechanism that sustains tumor growth and mediates resistance to gemcitabine, with inhibition strategies (e.g., chloroquine, autophagy blockers) shown to restore drug sensitivity in models.^353^ Thus, the hallmarks of PDAC involve profound metabolic rewiring driven by oncogenic mutations, autophagy, and macropinocytosis, which causes therapy resistance, immune evasion, and CSC plasticity in nutrient-deficient ME.

## Current and emerging targeted therapies

### Current therapies

Approximately 10 to 15% of PDAC cases are localized, and surgery remains the only option. Marginal invasive techniques, like laparoscopic and robotic pancreaticoduodenectomy, have become popular and demonstrate comparable survival outcomes, although evidence is largely observational.^354^ Despite developments in surgical expertise, including vascular resection and reconstruction, short-term morbidity such as pancreatic fistula and delayed gastric emptying remains a concern with a limited survival rate in the long term, and high recurrence driven by micro-metastatic disease during surgery.^355^ In localized, resectable PDAC, adjuvant chemotherapy improves survival compared to surgery alone. The most effective schedule for inpatients (ECOG 0–1) is adapted FOLFIRINOX fluorouracil/5-FU, oxaliplatin, irinotecan, and leucovorin, also supported by the PRODIGE-24 trial, with a median overall survival of gemcitabine.^356^ In PDAC with compromised response, the advised therapy is gemcitabine alone or in combination with gemcitabine plus capecitabine.^357^ Other routines include the use of the CONKO-001 trial, in which gemcitabine moderately improved median overall survival.^358^ Likewise, the ESPAC-1 trial presented that adjuvant 5-FU/leucovorin improved mOS by 5 months.^359^ A Phase II trial combination of gemcitabine with cisplatin has a higher mOS of 36 months, but was limited by high toxicity. The addition of erlotinib to gemcitabine was not beneficial in the adjuvant setting.^360^ The APACT experimental study investigated gemcitabine with nab-paclitaxel (GemNabP), which had no statistical difference initially, but a later analysis revealed a survival advantage.^361^ Thus, these data disclose that adjuvant chemotherapy improves disease-free and overall survival, with modified FOLFIRINOX remaining the preferred regimen for patients with better response, while gemcitabine-based combinations serve as effective alternatives for patients with reduced tolerance.^362,363^ A study demonstrates that in PDAC, neoantigen quality, not quantity, is associated with patient survival, with shorter survival linked to neoantigens exhibiting low similarity-to-self, derived mainly from a higher burden of INDELs rather than SNVs.^364^ While elevated INDEL frequency does not improve survival in untreated patients, it may predict improved responsiveness to ICB. Moreover, plasma cell infiltration provides CD8⁺ T cells with independent immunity in PDAC. Patient-derived organoids capture a significant fraction of tumor neoantigens, with potential utility for predicting survival and immunotherapy response, though validation in larger cohorts is required.

### Mutation-focused therapeutic approaches with a target for oncogenic pathways

In PDAC, the genetic alterations include ~94% KRAS mutations that drive constitutive oncogenic signaling through RAF-MEK-ERK and PI3K-AKT; ~64% TP53 mutations; ~98% CDKN2A mutations; and ~50% SMAD4 mutations, respectively, contributing to disruption of cell cycle control, apoptosis, and TGF-β signaling.^262^ Germline mutations in DNA repair mechanism regulators BRCA1/2, PALB2, and ATM additionally predispose to PDAC. The targeted therapy aims to exploit the vulnerabilities of mutations and signal transduction pathways.^365^ In KRAS mutant tumor cells, blockade by small molecules, deltarasin;^366^ MRTX1133, a KRAS G12D blockade, has reached phase I/II trials.^367^ Along with this, in the Phase I trial sotorasib (NCT03600883)a KRAS G12C inhibitor, is under study.^368^ Further strategies comprise knockdown of KRAS transcription by CRISPR-Cas13a or altered exosomes^346,369^ and inhibition of downstream effectors signal through MEK, PI3K, or RAF inhibitors. The PI3K blockade involving wortmannin,^370^ LY294002^371^ (BKM120) buparlisib^372^ ZSTK474, and GDC-0941^373^ demonstrated proapoptotic and antioncogenic activity, whereas the AKT inhibitor MK-2206^374^ enhanced efficacy in combination with chemotherapy. The dual PI3K/mTOR inhibitors NVP-BEZ235^375^ and urolithin A efficiently suppressed PDAC progression in engineered models. The MEK inhibitors AZD6244 (selumetinib),^376^ trametinib,^377^ and RO5126766,^378^ a RAS/MEK inhibitor, displayed synergistic antioncogenic activity in combination with PI3K inhibitors or standard chemotherapy. Loss of CDKN2A increases susceptibility to microtubule targeting agents like paclitaxel,^379^ while SMAD4-deficient tumors may benefit from TGF-β/SMAD signaling modulation as stromal barriers restrict drug and immune cell penetration, as TGF-β/SMAD signaling causes fibrosis and immune suppression. Enamine N-oxide–modified nanoparticles encapsulating a gemcitabine prodrug (GemC18) and the TGF-β/SMAD inhibitor galunisertib^380^ are effective.^381^ Thus, PDAC therapy is a mutation-driven approaches that integrate oncogenic pathway inhibition and DNA repair targeting.

## Emerging targeted therapies

In PDAC, targeted therapy, immunotherapy, and CAR-T cell approaches are emerging strategies to improve outcomes. Targeted therapies target specific mutations or signal pathways. Approved drugs for mutation-driven oncogenesis include larotrectinib, entrectinib, and repotrectinib for NTRK fusions; pembrolizumab for MSI-high, dMMR; dabrafenib and trametinib for BRAF V600E mutations; selpercatinib for RET fusions; and fam-trastuzumab deruxtecan for HER2 overexpression.^382^ Though such mutations are rare in PDAC, with a frequency of only 1–2% of MSI-high/dMMR mutations. Most prominent is the KRAS mutation at G12D, which is common but untargetable.^216^ Sotorasib and the new drug adagrasib were approved by the FDA for KRAS G12C, while MRTX1133, RMC-9805, and the pan-RAS inhibitor RMC-6236 are in clinical trials.^383^ Determinations to target upstream KRAS activators SHP2, SOS1 inhibitors, and downstream signal cascade of RAF/MEK/ERK and PI3K/AKT/mTOR are still under study.^384^ Tyrosine kinase target EGFR inhibitors, including small molecule erlotinib and antibody nimotuzumab, FAK inhibitors with chemotherapy, and BTK inhibitors acalabrutinib used with checkpoint inhibitor pembrolizumab, are in clinical trials.^385^ Novel gene fusion targets include RET selpercatinib, pralsetinib, and NRG1- zenocutuzumab, seribantumab. The MTAP/PRMT/CDKN2A pathway is also under analysis in subsets of PDAC with 9p21 deletions.^386^ Additionally, PARP inhibitors olaparib and rucaparib have better response in survival and are increasingly used in maintenance therapy.^387^ Thus, a rapidly evolving therapeutic landscape and its impact on PDAC remain limited to small patient subsets and require further validation in clinical trials. Recently, it has been identified that Nociceptor neurons promote PDAC progression and cancer pain by signaling through CGRP–NGF to suppress IL-15 expression in CAFs, thereby impairing NK cell infiltration and cytotoxicity. Nociceptive innervation has emerged as an independent prognostic marker and therapeutic target in PDAC.^388^ CAR-macrophage (CAR-M) therapy has been identified significant in PDAC by overcoming stromal and immune barriers through effective tumor infiltration, ME remodeling, improved antigen presentation, and recruitment of CD + 8 T cells, positioning CAR-Ms as a promising next-generation immunotherapy for resistant PDAC.^389^ Moreover, targeting the KRAS pathway through direct inhibitors, pathway modulation, siRNA, and rational combination strategies has overcome the “undruggable” status. Emerging alterations (NRG1, NTRK/ROS1, RET, PRMT5/CDKN2A/MAT2A), novel targets such as EGFR and Claudin18.2, and immunotherapy-based approaches for MSI-H PDAC, including adoptive cell therapies and cancer vaccines, collectively expand the therapeutic landscape and offer new precision medicine opportunities for PDAC.^228^ A study demonstrates that vitamin E–sphingomyelin lipid nanoemulsions reprogram pro-metastatic M2 tumor-associated macrophages toward an antitumor M1-like phenotype, suppressing PDAC growth and liver metastasis in human-relevant models, thus TAM reprogramming is a promising strategy to limit PDAC progression and metastasis.^390^ ALDH1A3 is a driver of the basal-like, therapy-resistant PDAC subtype, promotes tumor growth and poor survival by metabolic–epigenetic reprogramming that enhances histone H3 acetylation, AP-1 (FOS) activity, and oncogenic MAPK and TNF signaling, with RUNX2 emerging as a downstream therapeutic vulnerability capable of disrupting PDAC’s aggressive transcriptional program.^391^ PKMYT1 overexpression in PDAC is associated with poor outcomes. Inhibition of PKMYT1 by RP-6306 induces premature mitotic entry, DNA damage, mitotic catastrophe, and PANoptosis, thus suppressing tumor progression.^392^ RP-6306 synergizes with gemcitabine, though gemcitabine toxicity remains a concern. To enhance tumor specificity and safety, BxPC-3M, a cell membrane–derived vesicle, was utilized to co-deliver RP-6306 and gemcitabine.^393^ Thus, PANoptosis is a novel mitotic catastrophe-associated cell death mechanism and supports RP–6306–based combination therapy as a promising strategy for PDAC.^392^ Recent advances demonstrate that integrating genomic profiling with targeted therapies, immunomodulatory strategies, and TME–directed strategies such as novel KRAS inhibitors, CAR-T cells, immune checkpoint modulation, IL-6/CD137 targeting, cancer vaccines, and tumor treating fields may overcome therapeutic resistance and improve outcomes in PDAC.

## Conclusion and perspective

In summary, PDAC is a complex cancer with dismal outcomes. Although therapeutic strategies such as immunotherapy have enhanced survival outcomes in several cancers, they have not significantly improved PDAC prognosis. The major obstacle lies in the suppressive tumor microenvironment and the ECM of PDAC. Hence, a key unmet need exists for novel, efficient PDAC-ME-based therapeutic targets and biomarkers to improve drug efficacy and selectivity. Recent research indicates that multidrug combination therapy may be a promising approach to overcoming drug resistance and enhancing immunotherapy outcomes in PDAC. For instance, combining chemotherapy and radiotherapy with multiple immunotherapies, such as tumor vaccines and ICIs, can improve therapeutic efficacy against PDAC. Future research will delineate novel immunotherapeutic targets and treatment approaches for PDAC, leading to combinatorial approaches capable of overcoming resistance and improving outcomes.

PDAC development is driven by heterogeneous CAF subsets that remodel the stroma, suppress immunity, and interact with immune cells to stimulate tumor progression and resistance to therapy. The heterogeneity comprises proliferative, inflammatory, myofibroblastic, metabolic, senescent, and Ag-presenting CAFs that strongly reshape tumor ME, immune escape, metabolic reprogramming, and resistance to therapy. In PDAC, CAF heterogeneity is propelled by TME derived signals such as TGF-β, PDGF, FGF, inflammatory cytokines, metabolic and immunological signals, that activate interrelated pathways such as JAK/STAT, NF-κB, Wnt, Notch, Hedgehog, SMAD, MAPK, PI3K/AKT, to program distinct CAF phenotypes, increase desmoplasia, immune escape, metabolic reprogramming to support tumor progression, and resistance to therapy.

MDSCs are recognized as controllers of immune escape in PDAC by decreasing nonspecific and specific immune responses, enhancing angiogenesis, and strengthening crosstalk between the stromal-immune system. During arginine and cysteine depletion, ROS and NO generation, MDSCs weaken CD8 + T-cell activity and also intensify immunosuppression by inducing Tregs, TAMs, and Bregs. The functional modification into PMN-MDSCs and M-MDSCs enhances immune escape, with M-MDSCs controlling VEGF-dependent angiogenesis and metabolic immune suppression, and PMN-MDSCs inhibiting the T-cell effector role. Latest evidence emphasizes the novel TME-derived signaling axes that control MDSC recruitment, differentiation, and role in PDAC. Pathways like tumor-intrinsic regulators such as nerve-derived DKK1, TMBIM1, and CRIP1–NFκB–CXCL1/5 signaling together coordinate MDSC infiltration and spatial immune suppression. Inflammatory and lipid-based signals of PAF and tumor-derived lipid mediators stimulate neutrophil-to-PMN-MDSC differentiation, illuminating previously unrecognized metabolic inflammatory control of myeloid plasticity. IL-6, TGF-β, VEGF, GM-CSF, and STAT3 are cytokine-controlled pathways that integrate CAF–MDSC–TAM feedback loops, alleviating an immunosuppressive role and resistance to chemoimmunotherapy. The PDAC–MDSC axis represents a merging point of stromal signals (PD-L1, CD155–TIGIT), an immune checkpoint, angiogenic pathways, and metabolic stress, reduced antitumor immunity despite neoantigen presence. Emerging signals pathway identifies targetable vulnerabilities, supporting strategies that combine MDSC targeting with immune checkpoint blockade, CAF reprogramming, and CAR-T approaches, for example, CXCR4-CAR-T. Disrupting MDSC-mediated signaling networks thus holds strong translational potential to restore immune competence and overcome therapeutic resistance in PDAC.

The review discusses the DCs, which are essential to Ag- presentation and T-cell priming, are inadequately infiltrated and functionally impaired in PDAC due to stromal barriers, presence of inhibitory cytokines such as TGF-β and VEGF, and metabolic reprogramming. These factors influence suppression of DC maturation, antigen presentation, and co-stimulatory molecule expression, leading to ineffective activation of CD8 + T cells. Moreover, lipid accumulation, ER stress, and altered fatty acid metabolism further render DCs tolerogenic. DC-mediated vaccines and strategies reestablish the conventional DC role as Flt3L and CD40 agonists and are promising in reactivating CD8⁺ T-cell responses. However, refinement is needed to limit regulatory T-cell (Treg) expansion. T cells are also strongly affected by the PDAC TME. CD8⁺ T cells retain intrinsic antitumor potential but become exhausted due to inhibitory cytokines, tumor-intrinsic defect in Ag- presentation. CD4⁺ T cells exhibit functional plasticity, with Th1 cells supporting antitumor immunity, whereas Th2 cells and Tregs promote immune dominance and fibrosis. Tregs are highly triggered in PDAC, conveying multiple immune checkpoints and metabolic enzymes, making them crucial but complex therapeutic targets. Strategies, such as CCR8-targeting, deplete tumor-resident Tregs while minimizing systemic toxicity. NK cells are also an antitumor subset but are strongly inhibited by CAF-derived signals, MDSCs, TGF-β, and tumor-intrinsic signal pathways. Developing NK-cell mediated therapies, like adoptive transfer and CAR-NK cells, pursue to overcome these barriers. Immune checkpoint in PDAC is also a pivotal cause of immune escape; TCR profiling has emerged as a prognostic biomarker for immunotherapy response. While ICI alone has limited efficacy in PDAC, utilizing combination strategies by integrating chemoradiotherapy, targeting metabolic hotspots, and TME remodeling offer promising opportunities to convert PDAC from an immunologically “cold” to an efficient immune-responsive tumor. This need is further emphasized by the low immunogenicity of PDAC, which limits effective T-cell priming and ME activation, resulting in ineffective responses to ICB. It will thus be important to address individual differences in tumor antigenicity to accurately stratify patient populations and select optimal ICI combinations for each patient. Given that the immune response is dynamic and constantly changing, establishing superior predictors of response to ICI treatment is paramount to enhance the application of ICIs in clinical PDAC treatment. Hence, integrating traditional strategies and cutting-edge approaches will improve patient outcomes and redefine the PDAC treatment landscape, paving the way for more personalized therapeutic solutions.

Genetic, epigenetic, and signaling heterogeneity, as well as chromosomal instability and copy number alterations, intensify oncogenic signaling and inactivate tumor suppressors, promoting metastasis and therapy resistance. Therapeutically, KRAS is considered undruggable. Emerging evidence on KRAS G12C inhibitors (sotorasib, adagrasib) and G12D inhibitors (MRTX1133), have remodeled PDAC treatment strategies, though effectiveness is restricted to molecularly defined subsets and limited by adaptive resistance. Combination strategies targeting compensatory pathways are under investigation to overcome feedback reactivation and improve durability. DDR deficiencies signify another targetable susceptibility. PDAC patients with BRCA1/2, PALB2, or ATM modification benefited by platinum-based chemotherapy and PARP inhibitors in homologous recombination–deficient. Dual targeting of DDR and oncogenic KRAS-mediated replication stress, as well as combined inhibition of PARP and HDACs, has shown promising preclinical synergy. Also, epigenetic treatments are developing as a dominant modulator of PDAC plasticity and drug resistance. Targeting EZH2, BET proteins, CDK9, p300/CBP, and HDACs can sensitize tumors to chemoradiotherapy. RNA-based therapeutics (siRNA, miRNAs) and biomarkers (GATA6, L1CAM), and circulating miRNA enable patient response to treatment selection. Thus, this review provides a comprehensive understanding of the recent progress in PDAC immunotherapy, highlights key challenges, and discusses emerging approaches designed to improve patient outcomes.
